# Liver Sinusoidal Endothelial Cells Promote Metabolic Dysfunction-associated Steatohepatitis Progression via Interleukin-1R1-mediated Chemokine Production Induced by Macrophage-derived Interleukin-1β

**DOI:** 10.1016/j.jcmgh.2025.101698

**Published:** 2025-12-02

**Authors:** Kenji Fukumoto, Hayato Hikita, Yoshinobu Saito, Yuki Makino, Kazumasa Soma, Seiya Kato, Yoichi Sasaki, Yuta Myojin, Katsuhiko Sato, Sadatsugu Sakane, Kazuhiro Murai, Yuki Tahata, Takahiro Kodama, Tomohide Tatsumi, Daisuke Motooka, Yoshiaki Kubota, Shogo Kobayashi, Hidetoshi Eguchi, Tetsuo Takehara

**Affiliations:** 1 Department of Gastroenterology and Hepatology, The University of Osaka Graduate School of Medicine, Osaka, Japan; 2 NGS Core Facility, Research Institute for Microbial Diseases, Osaka University, Osaka, Japan; 3 Department of Anatomy, Keio University School of Medicine, Tokyo, Japan; 4 Department of Gastroenterological Surgery, The University of Osaka Graduate School of Medicine, Osaka, Japan

**Keywords:** Anakinra, CXCL10, IL1R1, LSEC, Macrophage, MASH, Spatial Transcriptomics

## Abstract

**Background & Aims:**

Interleukin (IL)-1β is a key cytokine in hepatitis-related inflammation, but its role in metabolic dysfunction-associated steatotic liver disease (MASLD) or steatohepatitis (MASH) remains unclear. This study investigated IL-1β-mediated interactions in nonparenchymal liver cells to elucidate their contributions to pathological MASH progression.

**Methods:**

We used the THP-1 monocyte-derived macrophage line and TMNK-1 liver sinusoidal endothelial cell (LSEC) line for in vitro assays. Endothelial cell-specific Il1r1-knockout (Il1r1^ΔEC^) and systemic Cxcl10-knockout (Cxcl10^-/-^) mice were subjected to a Western diet (WD) to establish a MASH model. An additional WD-fed cohort received the IL1R1 antagonist anakinra during the final 4 weeks. RNA sequencing data from liver tissues from patients with MASLD and spatial transcriptomic analyses focusing on nontumor regions of MASH-related hepatocellular carcinoma samples were evaluated.

**Results:**

Liver levels of mature IL-1β were elevated in WD-fed mice compared with ND-fed mice. Il1r1 was highly expressed in LSECs, and Ccl2 and Cxcl10 expression were upregulated in LSECs under WD conditions. Palmitic acid inhibited autophagy in THP-1 macrophages, increasing IL-1β secretion. IL-1β enhanced CCL2 and CXCL10 expression in TMNK-1 LSECs via JNK activation. In Il1r1^ΔEC^ and Cxcl10^-/-^ mice, WD-induced inflammatory cell infiltration and fibrosis were attenuated, and anakinra produced similar effects. In human datasets, CCL2 and CXCL10 were upregulated in MASH livers and correlated with NAFLD activity scores. Spatial transcriptomics revealed a dominant periportal macrophage-to-LSEC IL1B–IL1R1 interaction that generates chemokine-enriched LSECs, forming inflammatory–fibrotic niches that facilitate immune cell recruitment.

**Conclusions:**

Macrophage-derived IL-1β promotes hepatic inflammation and fibrosis through IL1R1-dependent chemokine induction in LSECs, highlighting IL1R1 signaling as a therapeutic target in MASH.

## Graphical abstract

**Figure:**
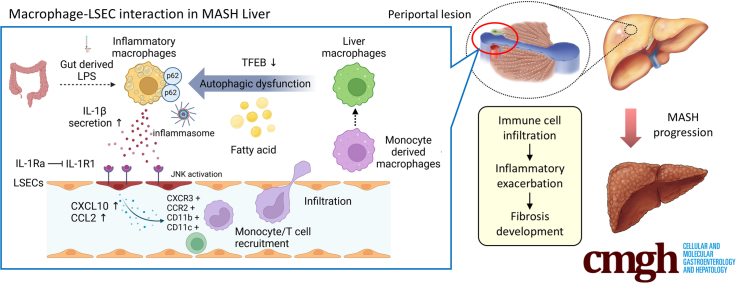

**Summary**

Macrophage-derived interleukin-1 beta activates liver endothelial cells to produce chemokines that recruit inflammatory cells and promote fibrosis. Spatial transcriptomic analysis revealed concentrated macrophage-to-endothelial signaling in periportal regions. Genetic and pharmacological blockade reduced steatohepatitis severity, highlighting this pathway as a therapeutic target.

**What You Need to Know**

**Background**

Interleukin (IL)-1β signaling contributes to hepatic inflammation, but how macrophage-derived IL-1β interacts with endothelial and stellate cells in metabolic dysfunction-associated steatohepatitis (MASH) remains poorly understood.

**Impact**

CosMx spatial transcriptomics identified macrophage-to-liver sinusoidal endothelial cell IL1B–IL1R1 signaling as a dominant interaction driving C-C motif chemokine ligand 2 and C-X-C motif chemokine ligand 10 induction, linking inflammation and fibrosis in MASH.

**Future Directions**

Targeting IL1R1 signaling may represent a promising therapeutic approach to mitigate hepatic inflammation and fibrotic progression in MASH patients.

Due to the global rise in the prevalence of obesity and type 2 diabetes, metabolic dysfunction-associated steatotic liver disease (MASLD) and metabolic dysfunction-associated steatohepatitis (MASH) have become the most common liver diseases, affecting approximately a quarter of the world’s population.^1^ MASH increases the risk of hepatocellular carcinoma (HCC) and is considered a systemic metabolic disorder that also increases the risk of cardiovascular complications.^2^ The pathophysiology of MASH involves not only lipotoxicity but also insulin resistance, alterations in the gut microbiota, and various interactions within the liver microenvironment.^3^^,^^4^ Understanding these complex mechanisms is crucial for developing effective strategies for the prevention and treatment of MASH.^5^

Interleukin (IL)-1β is a proinflammatory cytokine that plays a central role in the innate immune response. It is produced primarily by macrophages and is activated in response to the presence of pathogen-associated molecular patterns (PAMPs) and damage-associated molecular patterns (DAMPs).^6^ IL-1β exerts its effects through the IL-1 receptor (IL-1R), activating the nuclear factor kappa B (NF-κB) and mitogen-activated protein kinase (MAPK) signaling pathways to amplify inflammatory responses. IL-1β has been implicated in various pathological liver conditions, such as alcoholic liver disease (ALD),^7^ viral hepatitis (especially hepatitis C virus [HCV] infection),^8^ and acute liver failure (ALF).^9^ In ALD patients, lipopolysaccharides (LPS) derived from the gut microbiota activate liver macrophages, leading to increased IL-1β production, thereby accelerating liver damage and fibrosis. In patients with viral hepatitis, including HCV infection, viral components stimulate intrahepatic macrophages, promoting IL-1β secretion, which drives chronic inflammation and enhances fibrosis progression. Similarly, in patients with ALF, extensive hepatocyte necrosis releases DAMPs, excessively activating IL-1β signaling and contributing to uncontrolled inflammation and multiorgan failure.

In patients with MASH, alterations in the gut microbiota increase the production of LPS, which reaches liver macrophages via the portal vein and induces IL-1β secretion.^10^^,^^11^ Additionally, stimulation by fatty acids and other DAMPs activates the inflammasome in liver macrophages, further increasing IL-1β production and triggering hepatitis.^12^^,^^13^ However, the precise mechanisms underlying increased IL-1β secretion in patients with MASH, the specific IL-1β target cells, and the downstream liver pathology effects remain unclear. Recent advancements in single-cell analyses have allowed researchers to gradually elucidate the roles of intrahepatic nonparenchymal cells (NPCs) in MASH, highlighting the importance of intercellular interactions.^14^^,^^15^ Given the established role of IL-1β in various liver diseases, IL-1β likely contributes to MASH progression by interacting with intrahepatic NPCs to promote inflammation and fibrosis. In this study, we aimed to explore how IL-1β-mediated intercellular interactions influence the pathogenesis of MASH and contribute to disease progression.

## Results

### Feeding Mice a Western Diet Increases IL-1β Levels and Impairs Macrophage Autophagy in the Liver

**Figure 1:**
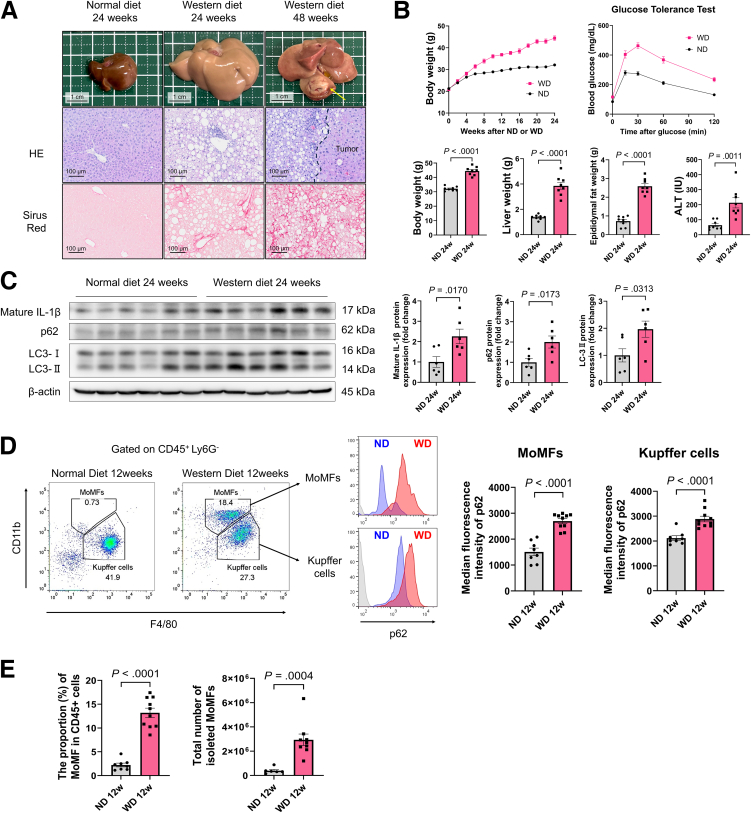
**WD-induced MASH impairs autophagy in hepatic macrophages and increases mature IL-1β levels in liver tissue.** (*A*) Histological analysis of liver tissue from a mouse model of MASH induced by ND or WD administration. (*B*) Phenotypic characteristics of mice fed an ND or WD (n = 8/group), with a GTT conducted at 24 weeks (n = 12/group). (*C*) Mature IL-1β and autophagy-related proteins in the liver tissues of mice were evaluated using Western blotting. (*D*) The median fluorescence intensity (MFI) of p62 in liver macrophages (MoMFs and KCs) from mice fed an ND or WD for 12 weeks (n = 8–10 mice/group) was analyzed using flow cytometry. (*E*) (*Left panel*) The proportion of MoMFs among CD45-positive liver cells was compared between ND- and WD-fed mice (n = 8–10 mice/group). (*Right panel*) The total number of isolated MoMFs was calculated based on the yield of NPCs obtained from each liver (n = 6–9 mice/group).

Mice fed a Western diet (WD) presented pathological features of MASH, including fat accumulation, inflammatory cell infiltration, and liver fibrosis. Approximately 40% of these mice (7/18) developed tumors after 48 weeks (Figure 1*A*). They also exhibited weight gain, impaired glucose tolerance, and increased serum alanine aminotransferase (ALT) levels and were considered a physiological model closely resembling human MASH (Figure 1*B*). Pro-IL-1β is cleaved into its mature form, enabling its secretion. Compared with those in the normal diet (ND) group, the mature liver tissue IL-1β protein levels were increased in mice after 24 weeks of WD feeding (Figure 1*C*). In the WD group, increased levels of the autophagy-related proteins p62 and LC3-II were observed (Figure 1*C*), suggesting autophagy impairment-induced accumulation, which is consistent with previous reports.^16^

**Figure 3:**
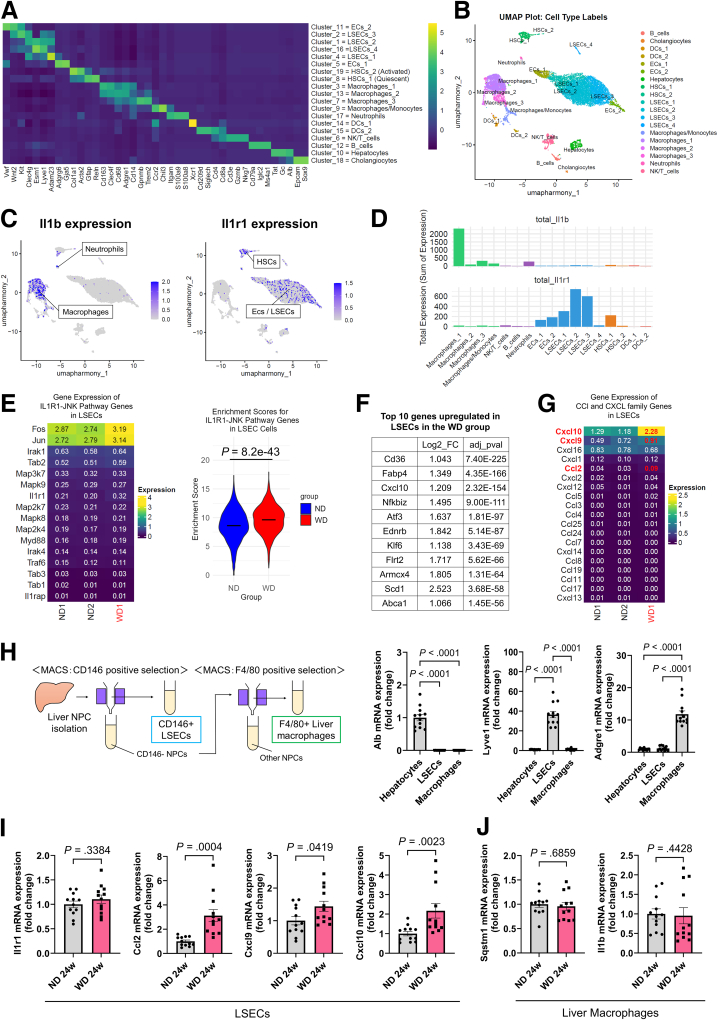
**Inflammatory chemokine expression is upregulated in the LSECs of WD-fed mice.** (*A–G*) Single-cell RNA-seq of liver tissues from wild-type mice fed an ND (n = 2) and a WD (n = 1). (*A*) A heatmap of clusters and representative marker genes used for cell type identification. (*B*) Cell types according to UMAP. (*C*) The gene expression of *Il1b* and *Il1r1* on the UMAP, combining the ND and WD groups. (*D*) A bar graph showing the total *Il1r1* or *Il1b* expression summed across all cells of each cell type in NPCs from the WD group. (*E*) A heatmap comparing the expression levels of genes listed in the KEGG IL1-IL1R-JNK signaling pathway (N00188) in LSECs between samples (*left*), and a violin plot comparing the enrichment scores calculated based on the total expression levels of those genes between groups (*right*). The bars on the plot represent the mean. (*F*) Top 10 upregulated genes in LSECs in the WD group compared with the ND group ranked by *P* value. (*G*) A heatmap of inflammatory chemokine gene expression in LSECs of each mouse. (*H*) Isolation of LSECs and macrophages from ND-fed mice liver by MACS and purity assessment by RT-qPCR (n = 12/group). (*I* and *J*) Gene expression in isolated LSECs (*I*) and liver macrophages (*J*) (n = 12/group).

To evaluate autophagy in hepatic macrophages, a primary source of IL-1β, we measured p62 expression levels using flow cytometry. The macrophages were classified as monocyte-derived macrophages (MoMFs) (CD45^+^ Ly6G^-^ Cd11b^high^ F4/80^+^) or Kupffer cells (KCs) (CD45^+^ Ly6G^-^ Cd11b^int^ F4/80^+^). The mean fluorescence intensities of p62 expression were significantly greater in both MoMFs and KCs from livers of WD-fed mice than in those from ND-fed mice (Figure 1*D*). Conversely, the expression of the *Sqstm1* gene, which encodes p62, in liver macrophages isolated from ND- and WD-fed mice did not differ (Figure 3*J*), suggesting that WD feeding led to impaired autophagy in macrophages. The proportion of MoMFs among hepatic CD45^+^ cells was significantly higher in WD-fed mice than in ND-fed mice. In addition, the absolute number of isolated hepatic MoMFs was also increased in WD-fed mice, indicating increased recruitment and expansion of MoMFs under MASH conditions (Figure 1*E*).

### Palmitic Acid Promotes IL-1β Secretion by Impairing Macrophage Autophagy

**Figure 2:**
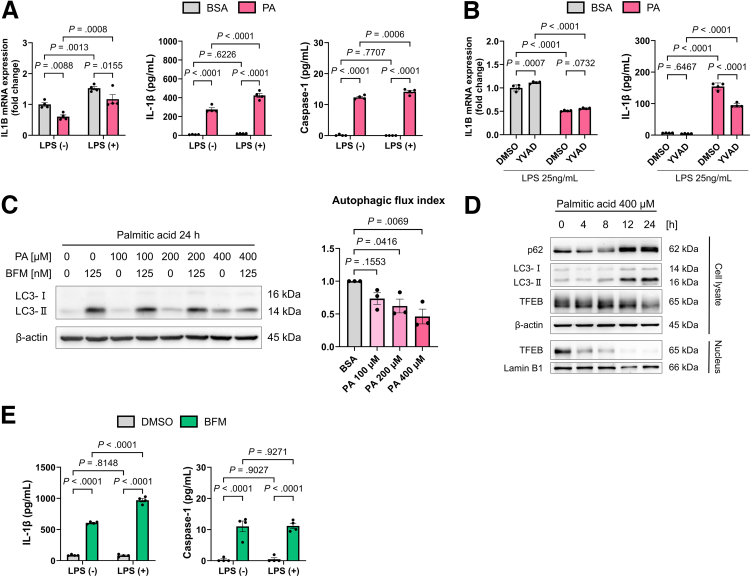
**The impairment of autophagy in macrophages by fatty acids increases IL-1β secretion.** (*A*) IL1B gene expression and the concentrations of IL-1β and caspase-1 in the supernatants of THP-1 macrophages cultured for 24 hours with or without LPS (25 ng/mL) and treated with either BSA or 400 μM PA (n = 4/group). (*B*) *IL1B* gene expression and the concentrations of IL-1β in the supernatants of THP-1 macrophages cultured for 24 hours with BSA or 400 μM PA and 25 ng/mL LPS in the presence or absence of 50 μM Z-YVAD-FMK (n = 4/group). (*C*) LC3 turnover assay to evaluate autophagic flux in THP-1 macrophages treated with PA and BFM (n = 3/group). (*D*) Expression of autophagy-related proteins in THP-1 macrophages treated with PA. (*E*) IL-1β and caspase-1 concentrations in the supernatants of THP-1 macrophages cultured for 24 hours with or without 25 ng/mL LPS and treated with either dimethyl sulfoxide (DMSO) or 50 nM BFM.

Next, we investigated the effects of palmitic acid (PA) administration on macrophages in vitro, as PA is a key fatty acid involved in MASH pathophysiology. When PA was added to LPS-stimulated THP-1 macrophages, *IL1B* gene expression did not increase, but IL-1β secretion was increased (Figure 2*A*). IL-1β is cleaved from pro-IL-1β upon inflammasome activation.^6^ Caspase-1 secretion, a marker of inflammasome activation, increased with PA treatment (Figure 2*A*). The PA-induced increase in IL-1β secretion, without changes in *IL1B* gene expression, was attenuated by treatment with Z-YVAD-FMK, a caspase-1 inhibitor (Figure 2*B*).

We investigated the involvement of autophagy in PA-induced inflammasome activation. In patients with acute hepatitis, autophagy regulates inflammation by degrading excessive inflammasomes and reactive oxygen species (ROS).^17^ The addition of PA to THP-1 macrophages decreased autophagic flux, with a decrease in transcription factor EB (TFEB) levels and increases in p62 and LC3-II protein levels (Figure 2*C* and *D*), suggesting the impairment of late-stage autophagy. TFEB is a critical protein for lysosomal biogenesis,^18^^,^^19^ and reduction of its expression suggests autophagy dysfunction. Treatment with the autophagy inhibitor bafilomycin (BFM) also increased IL-1β secretion and caspase-1 release (Figure 2*E*). These findings suggest that fatty acid-induced autophagy dysfunction enhances macrophage IL-1β secretion through inflammasome activation.

### Inflammatory Chemokine Expression Is Upregulated in the LSECs of Experimental MASH Mice

The single-cell analysis of the livers of wild-type mice fed an ND (n = 2) or a WD (n = 1) for 12 weeks revealed 19 cell clusters, including 4 macrophage clusters (Figure 3*A*, *B*). *Il1b* was highly expressed in macrophages and neutrophils, whereas its receptor, *Il1r1*, was highly expressed in liver sinusoidal endothelial cells (LSECs) and hepatic stellate cells (HSCs) (Figure 3*C*). Based on the total expression levels in WD-fed mice, in which *Il1b* expression was highest in macrophages and *Il1r1* expression was highest in LSECs (Figure 3*D*), we focused on the interaction between these cells. In LSECs, the total expression levels of genes listed in the Kyoto Encyclopedia of Genes and Genomes (KEGG) IL1-IL1R- c-Jun N-terminal kinase (JNK) signaling pathway (N00188) were calculated, and the enrichment scores were compared. The results revealed higher scores in the WD group than in the ND group (Figure 3*E*). In the cells of the LSEC clusters (LSEC 1–4), the top 10 genes with the lowest adjusted *P* values whose expression was upregulated by more than 2-fold in the WD group compared with that of the ND group included the lipid metabolism-related genes *Cd36* and *Fabp4*, as well as the inflammatory chemokine *Cxcl10* (Figure 3*F*). The expression levels of the inflammatory chemokines *Cxcl10*, *Cxcl9*, and *Ccl2* were increased among Cxcl and Ccl family genes (Figure 3*G*). LSECs were isolated from mouse liver tissues using magnetic-activated cell sorting (MACS) to validate the increased expression levels of these chemokines (Figure 3*H*). Although no difference in *Il1r1* gene expression was observed between groups, the expression of the *Ccl2*, *Cxcl9*, and *Cxcl10* genes was confirmed to be upregulated in the LSECs isolated from the WD group compared with those from the ND group (Figure 3*I*).

### IL-1β From Macrophages Increases CCL2 and CXCL10 Expression in LSECs Through JNK Activation

**Figure 4:**
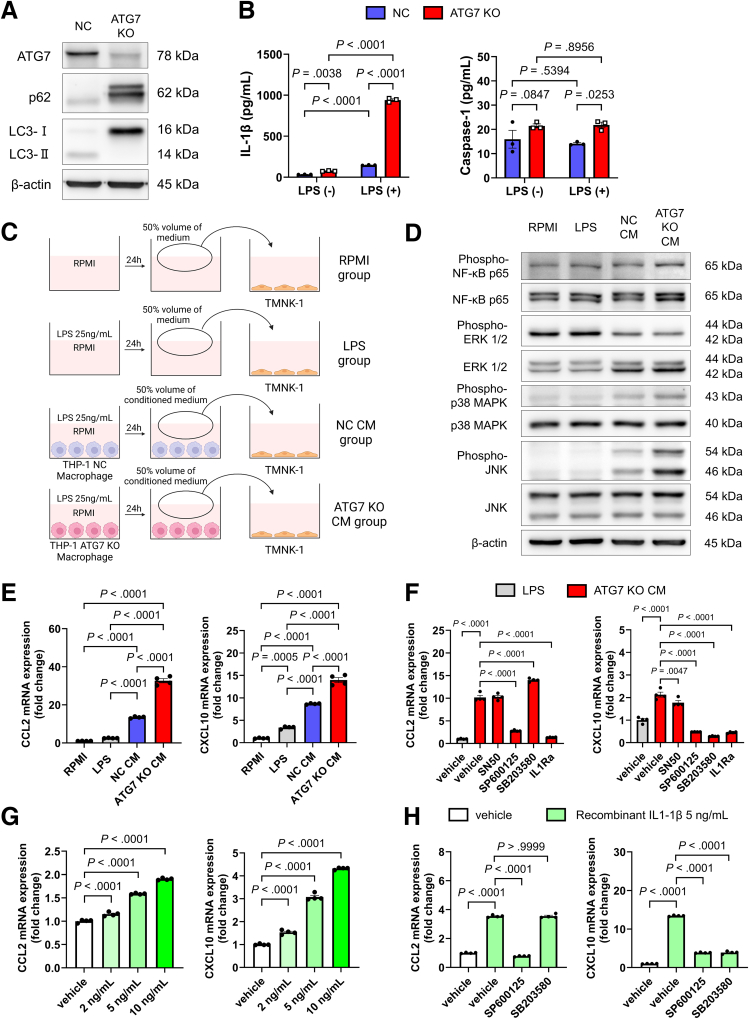
**Increased IL-1β secretion by macrophages due to autophagy impairment enhances inflammatory chemokine expression in LSECs.** (*A*) Protein expression in ATG7-KO macrophages and NC macrophages generated using the CRISPR-CAS9 system. (*B*) Evaluation of IL-1β and caspase-1 levels in the culture supernatant of THP-1 macrophages by ELISA following 24-hour stimulation with or without 25 ng/mL LPS (n = 3/group). (*C*) Experimental method using LPS-stimulated THP-1 macrophage conditioned medium. (*D*) Evaluation of NF-κB or MAP kinase-related proteins in TMNK-1 cells. (*E* and *F*) Inflammatory chemokine gene expression in TMNK-1 cells when treated with control medium or THP-1 macrophage conditioned medium (n = 4/group). SN50, NF-κB inhibitor; SP600125, JNK inhibitor; SB203580, p38 MAPK inhibitor. (*G*) Inflammatory chemokine gene expression measured 24 hours after treating TMNK-1 cells with varying concentrations of human recombinant IL-1β (n = 4/group). (*H*) Evaluation of inflammatory chemokine expression in TMNK-1 cells after 6 hours of stimulation with recombinant human IL-1β (5 ng/mL) in the presence of SP600125 and SB203580 (n = 4/group).

Next, we investigated macrophage–LSEC interactions in vitro, focusing on their roles in MASH pathogenesis. Because impaired macrophage autophagy is thought to occur in individuals with MASH (Figures 1*D*, 2*C* and 2*D*), we first generated THP-1 cells in which the autophagy gene *ATG7* was knocked out (Figure 4*A*). Similar to the effects of PA and BFM administration (Figures 2*A*, 2*B* and 2*E*), the ATG7 knockout (KO)-induced autophagy disruption led to increased IL-1β secretion in THP-1 macrophages, along with increased caspase-1 secretion (Figure 4*B*). Human LSEC (TMNK-1) cells were cultured with the conditioned medium from LPS-stimulated THP-1 macrophages to assess the effect of macrophage-derived soluble factors on LSECs (Figure 4*C*). The conditioned medium from macrophages increased the expression of *CCL2* and *CXCL10* in TMNK-1 cells and activated p38 MAPK and JNK (Figure 4*D* and *E*). These effects were further enhanced when ATG7 expression was knocked out in macrophages (Figure 4*D* and *E*). The increased expression of *CCL2* and *CXCL10* in TMNK-1 cells cultured with ATG7-KO macrophage conditioned medium was markedly suppressed by the addition of an IL-1 receptor antagonist (IL-1Ra) (Figure 4*F*). These findings suggest that IL-1β, whose secretion was increased due to impaired autophagy induced by macrophage-conditioned medium, is the main driver of the increased chemokine expression in TMNK-1 cells. Furthermore, administration of a JNK inhibitor suppressed the expression of both *CCL2* and *CXCL10*, whereas the administration of a p38 MAPK inhibitor specifically suppressed *CXCL10* gene expression, indicating that the JNK and p38 MAPK pathways downstream of IL1R1 are involved (Figure 4*F*). Recombinant human IL-1β was added to TMNK-1 cells to further investigate this pathway, and increased expression of the *CCL2* and *CXCL10* genes was observed (Figure 4*G*). This effect was suppressed by administration of the JNK and p38 MAPK inhibitors (Figure 4*H*).

**Figure 5:**
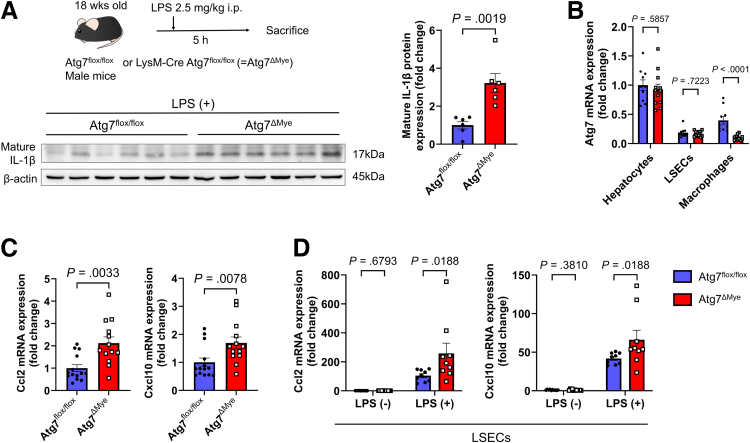
**Myeloid-specific Atg7 deficiency is associated with increased levels of mature IL-1β in liver tissue and increased chemokine expression in LSECs.** (*A*) Evaluation of mature IL-1β protein levels in liver tissues from Atg7^flox/flox^ or Atg7^ΔMye^ mice in LPS intraperitoneal injection model mice (n = 6/group). (*B*) Atg7 gene expression in cells isolated from liver tissues of Atg7^flox/flox^ or Atg7^ΔMye^ mice (n = 10–12/group). (*C*) Expression of inflammatory chemokine genes in liver tissues from model mice intraperitoneally injected with LPS (n = 13–14 mice/group). (*D*) Expression of inflammatory chemokine genes in isolated LSECs (n = 9–12/group).

Mice with bone marrow-derived cell-specific Atg7 KO (LysM-Cre Atg7^flox/flox^ [Atg7^ΔMye^] mice) or control mice (Atg7^flox/flox^ mice) were intraperitoneally injected with LPS to examine the effect of impaired macrophage autophagy on *CCL2* and *CXCL10* expression in LSECs in vivo (Figure 5*A*). Atg7^ΔMye^ mice presented decreased *Atg7* expression in liver macrophages but no changes in its expression in LSECs (Figure 5*B*). Compared with Atg7^flox/flox^ mice, Atg7^ΔMye^ mice presented increased IL-1β levels in the liver (Figure 5*A*). The expression levels of *Ccl2* and *Cxcl10* were higher in the whole liver and LSECs from Atg7^ΔMye^ mice than in those from the Atg7^flox/flox^ mice (Figure 5*C* and *D*).

### Endothelial Cell-specific *Il1r1*-KO Alleviates Liver Fibrosis and Inflammatory Cell Infiltration in MASH-affected Livers and Reduces *CCL2* and *CXCL10* Expression in LSECs

**Figure 6:**
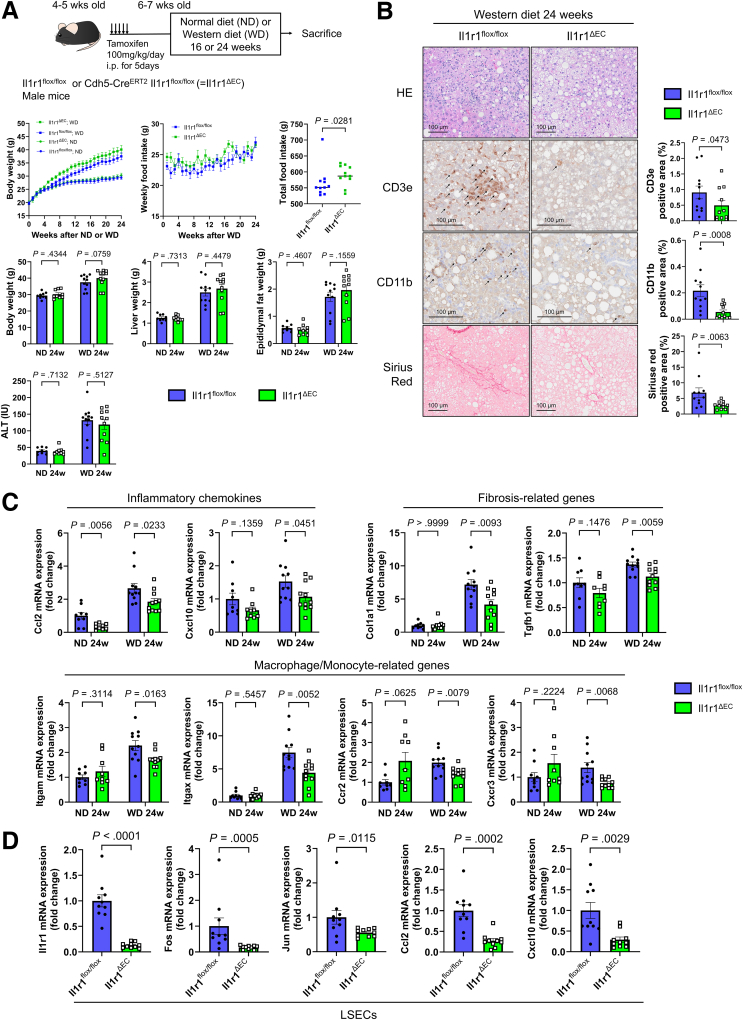
**EC-specific Il1r1 KO improves MASH pathology progression induced by a WD.** (*A*) Phenotype and WD intake of Il1r1^flox/flox^ or Il1r1^ΔEC^ mice (n = 9–11/group). (*B*) Histological evaluation of liver tissues in the WD group (n = 11/group). (*C*) Inflammation and fibrosis-related gene expression in liver tissues (n = 9–11/group). (*D*) Gene expression in isolated LSECs after 16 weeks of WD feeding (n = 10/group).

To investigate the impact of IL-1β on MASH pathogenesis via LSECs, we fed a WD to tamoxifen-inducible endothelial cell (EC)-specific Il1r1-KO mice (Cdh5-Cre^ERT2^ Il1r1^flox/flox^, Il1r1^ΔEC^). Compared with Il1r1^flox/flox^ mice, Il1r1^ΔEC^ mice consumed more food and tended to weigh more (Figure 6*A*). No significant difference in ALT levels was observed between groups (Figure 6*A*), but immunohistochemical staining showed that the CD3e- and CD11b-positive liver areas were reduced in Il1r1^ΔEC^ mice (Figure 6*B*). Additionally, the expression levels of *Ccl2*, *Cxcl10*, and monocyte/macrophage-related genes in liver tissues were lower in Il1r1^ΔEC^ mice than in Il1r1^flox/flox^ mice (Figure 6*C*). Moreover, the Sirius red-positive area and the expression of fibrosis-related genes in liver tissues were reduced in Il1r1^ΔEC^ mice (Figure 6*B*), indicating the attenuation of inflammation and fibrosis in experimental MASH mice.

*Il1r1* expression was suppressed in LSECs isolated from Il1r1^ΔEC^ mice using MACS (Figure 6*D*). Additionally, the expression levels of *Ccl2* and *Cxcl10* were lower in Il1r1^ΔEC^ mice than in Il1r1^flox/flox^ mice (Figure 6*D*). The expression of genes related to the IL-1β–IL1R1–JNK pathway, such as *Fos* and *Jun*, was reduced in the Il1r1^ΔEC^ mice (Figure 6*D*), suggesting that this reduction may be involved in the downregulation of inflammatory chemokine expression.

### Cxcl10-KO Alleviates Liver Fibrosis and Inflammatory Cell Infiltration in MASH-affected Livers

**Figure 7:**
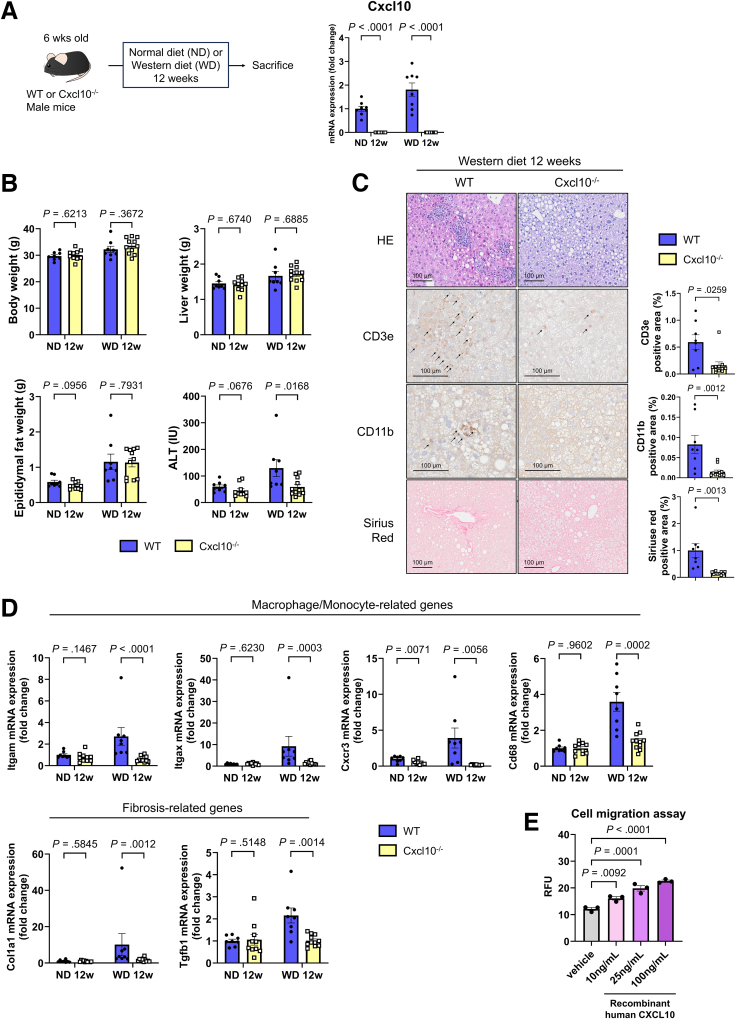
**Systemic Cxcl10 KO improves MASH pathology progression induced by a WD.** (*A*) Cxcl10 gene expression in liver tissues (n = 8–11/group). (*B*) Phenotype of WT or Cxcl10^-/-^ mice (n = 8–11/group). (*C*) Histological evaluation of liver tissue in the WD group (n = 8–11/group). (*D*) Macrophage/monocyte and fibrosis-related gene expression in liver tissues (n = 8–11/group). (*E*) A cell migration assay was performed on THP-1 cells using a CytoSelect 24-well cell migration assay (catalog number 093-05991; FUJIFILM Wako Pure Chemical Corporation). THP-1 cells were placed in the upper chamber, and after adding human recombinant CXCL10 at various concentrations, the cells were incubated for 24 hours. The cells that migrated to the lower chamber were dissolved with a specified reagent, and absorbance was measured (n = 3/group). RFU, relative fluorescence unit.

To investigate the impact of Cxcl10 signaling on MASH pathogenesis, we fed a WD to systemic Cxcl10-KO mice (Cxcl10^-/-^ mice). The expression levels of *Cxcl10* were completely inhibited even after WD feeding (Figure 7*A*). After WD feeding, Cxcl10^-/-^ mice presented no differences in body weights or liver weights, but their ALT levels were lower than those of wild-type mice (Figure 7*B*). Compared with those in wild-type mice, the CD3e- and CD11b-positive liver areas were reduced in Cxcl10^-/-^ mice (Figure 7*C*). The hepatic expression levels of macrophage/monocyte-related genes were lower in Cxcl10^-/-^ mice than in WT mice (Figure 7*D*). Additionally, the Sirius red-positive area and the expression of fibrosis-related genes in liver tissues were also decreased in Cxcl10^-/-^ mice (Figure 7*C* and *D*).

To examine the effect of Cxcl10 on monocytes, we administered recombinant human CXCL10 to THP-1 cells. Recombinant human CXCL10 increased the migratory ability of THP-1 cells, suggesting that CXCL10 may mobilize monocytes (Figure 7*E*). These findings suggest that LSEC-derived CXCL10 may contribute to the progression of MASH pathology by recruiting immune cells, including monocytes.

### IL1R1 Blockade With Anakinra Attenuates Hepatic Inflammation and Fibrosis in Mice With Established MASH

**Figure 8:**
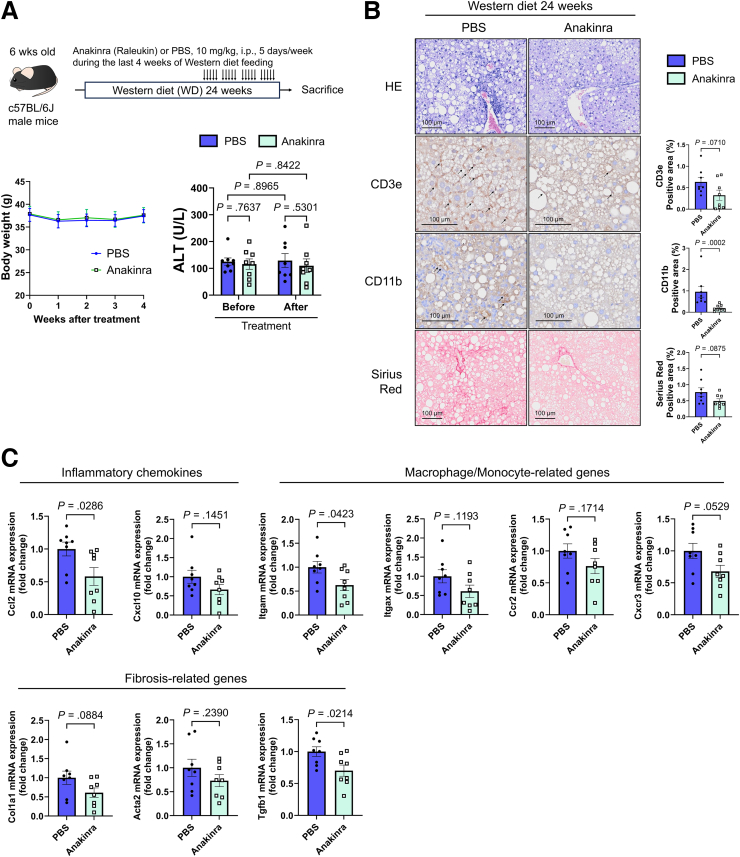
**Anakinra exerts potential therapeutic effects on a MASH mouse model.** (*A*) Therapeutic schedule of anakinra treatment in the MASH mouse model and changes in body weight and serum ALT levels before and after treatment. (*B*) Histological evaluation of liver tissues (n = 8 mice/group). (*C*) Inflammation and fibrosis-related gene expression in liver tissues (n = 8 mice/group).

We administered the IL1R1 antagonist anakinra to mice during the final 4 weeks of WD feeding to further evaluate the therapeutic relevance of IL-1β–IL1R1 signaling in established MASH (Figure 8*A*). No differences in body weight or baseline serum ALT levels were observed between groups prior to treatment. After treatment, anakinra-treated mice showed modest reductions in CD11b- and CD3e-positive areas in the liver, accompanied by a trend toward decreased Sirius red-positive fibrosis (Figure 8*B*). Consistent with these histological findings, hepatic expression of inflammatory chemokine genes, including *Ccl2* and *Cxcl10*, and monocyte/macrophage-related genes such as *Itgam*, was reduced following anakinra treatment (Figure 8*C*). The expression of fibrosis-related genes, including Tgfb1, was also downregulated (Figure 8*C*). These results support the hypothesis that IL1R1 signaling contributes to immune cell recruitment and fibrotic progression during MASH and suggest that pharmacological IL1R1 blockade may represent a potential therapeutic strategy.

### The Expression Levels of Inflammatory Chemokines Are Increased in the Livers of Patients With MASH

**Figure 9:**
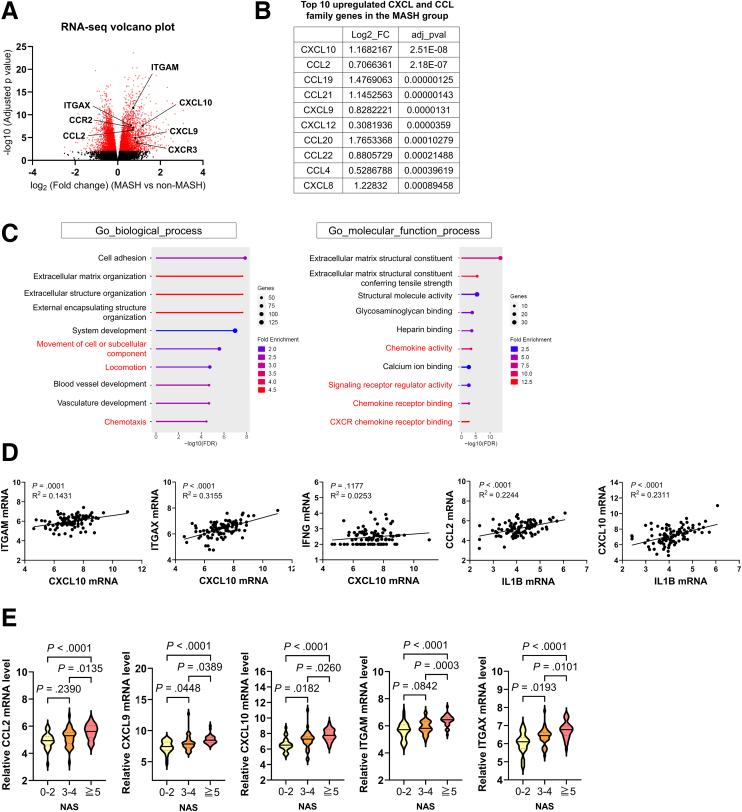
**Macrophage-LSEC-mediated CXCL10 induction is involved in the progression of human MASH pathology.** (*A*–*E*) RNA-seq analysis of 98 patients with MASLD (GSE167523). (*A*) Volcano plot and enrichment analysis of differentially expressed genes (DEGs) based on RNA-seq. (*B*) Top 10 upregulated CXCL and CCL family genes in the MASH group compared with the non-MASH group, ranked by *P* value. (*C*) DEG enrichment analysis plots using Gene Ontology (GO) biological process (*left*) and GO molecular function process (*right*). The pathway names related to chemokines and cell migration are highlighted in *red*. (*D*) Correlation plot of inflammation-related gene expression levels. (*E*) Expression of inflammation-related genes by NAFLD Activity Score. The *solid line* in the plot represents the median, and the *dashed lines* indicate the quartiles.

We investigated the correlation between the expression of CXCL10 and other inflammation-related genes and disease progression in patients with MASLD. The RNA sequencing data from 98 patients with MASLD (GSE167523), which we previously reported,^20^ revealed that expression of *CCL2* and *CXCL10*, as well as that of monocyte/macrophage marker genes such as *ITGAM* and *ITGAX*, was upregulated in the MASH group compared with the non-MASH group (Figure 9*A*). Furthermore, among the CCL and CXCL family members whose expression was upregulated in the MASH group, the top genes with the lowest adjusted *P* values were CXCL10 and CCL2 (Figure 9*B*). The enrichment analysis revealed that the activation of pathways related to chemokines and cell migration was upregulated in the MASH group (Figure 9*C*). The expression of the *ITGAM* and *ITGAX* genes was positively correlated with *CXCL10* expression (Figure 9*D*). The expression of these inflammation-related genes increased with increasing nonalcoholic fatty liver disease (NAFLD) activity scores (Figure 9*E*). These findings suggest that the increased expression of inflammatory chemokines, including CXCL10, and the increased infiltration of MoMFs play crucial roles in the progression of MASH pathology.

### Spatial Transcriptomics Identifies Periportal Macrophage–LSEC IL1B–IL1R1 Signaling as a Dominant Inflammatory–Fibrotic Interaction in Patients With MASH

**Figure 10:**
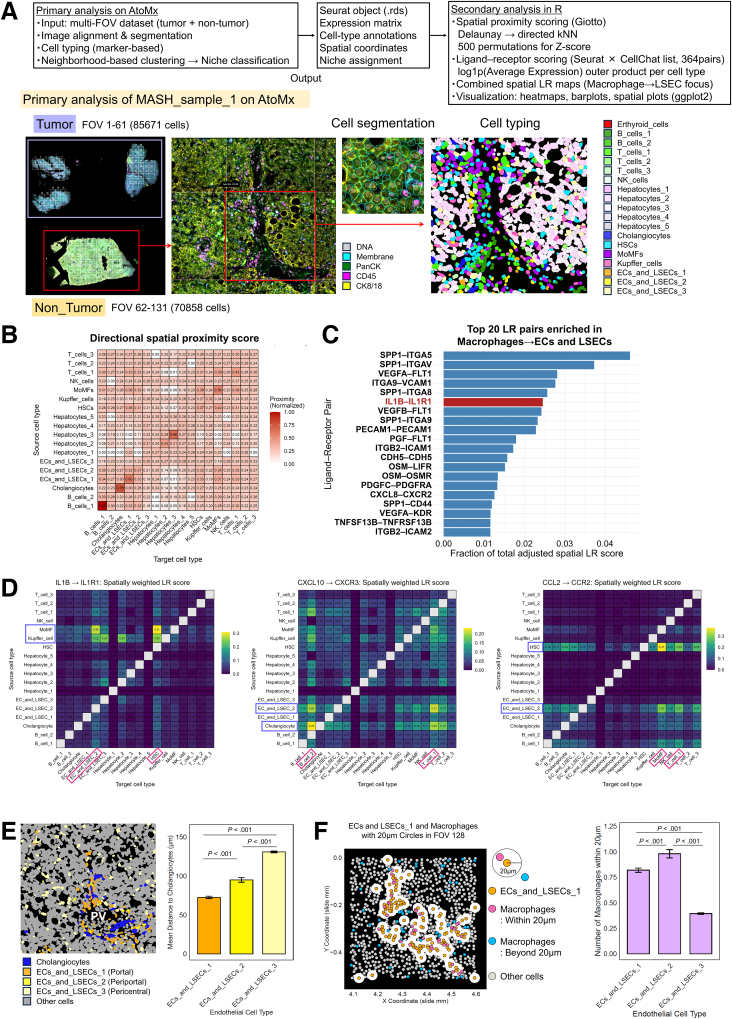
**Spatial transcriptomic analysis reveals a dominant IL1B–IL1R1 axis between macrophages and LSECs, with increased chemokine expression in periportal ECs accompanied by increased macrophage proximity.** (*A*) Workflow for the analysis of liver tissues from a patient with MASH (CosMx_MASH_sample_1) using CosMx. (*B*) Heatmap of the directional spatial proximity score across source-target cell type pairs. (*C*) Top 20 LR pairs enriched in macrophage → endothelial cell/LSEC interactions based on spatial LR scores. (*D*) Heatmaps of spatially weighted LR scores. From left to right: IL1B–IL1R1, CXCL10–CXCR3, and CCL2–CCR2. Higher values indicate the preferential enrichment of source→target interactions. (*E*) (*Left panel*) Positional relationship between each EC and cholangiocyte in the FOV. (*Right panel*) Comparison of the shortest distance from each EC to the nearest cholangiocyte. (*F*) (*Left panel*) Positional relationship between ECs and LSECs and macrophages in the FOV. The *white circles* represent a 20-μm radius from each EC. (*Right panel*) Comparison of the average number of macrophages within 20 μm of each EC.

We performed a high-resolution spatial transcriptomic analysis of surgically excised liver tissues from 2 patients with MASH-related HCC using CosMx to further characterize intercellular communication in MASH. In the first patient (CosMx_MASH_sample_1), cells were segregated into multiple major cell clusters, including 3 endothelial cell/LSEC subsets (ECs_and_LSECs_1–3) (Figure 10*A*). We first calculated directional spatial proximity scores between all cell type pairs to quantify the likelihood of physical adjacency (Figure 10*B*). Based on these proximity matrices, we then computed spatially weighted ligand-receptor (LR) scores for 364 one-to-one LR pairs by integrating ligand/receptor expression levels with source→target proximity. Among all cell type combinations, LR pairs enriched in macrophage→endothelial cell/LSEC interactions were identified, and IL1B–IL1R1 emerged as one of the top enriched pairs (Figure 10*C*). Spatial LR heatmaps further showed that IL1B–IL1R1 signaling was particularly pronounced from macrophages to the ECs_and_LSECs_2 subset (Figure 10*D*). Notably, this endothelial subset expressed CCL2 and CXCL10 at high levels, similar to HSCs and cholangiocytes, suggesting a potential role in recruiting MoMFs, T cells, and B cells.

**Figure 11:**
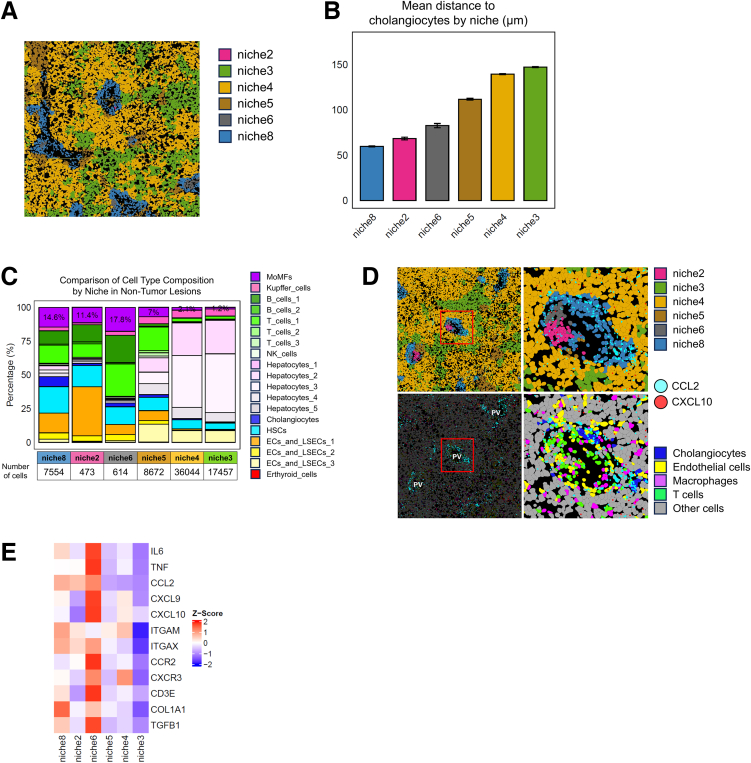
**Periportal niches enriched for inflammation and fibrosis in MASH liver tissue.** (*A*) Image of the FOV with the non-tumor regions color-coded by niche. (*B*) Bar plot of the mean distance to cholangiocytes by niche. (*C*) Bar plot showing the proportions of cell types in each niche of the non-tumor region. (*D*) Images of the FOV color-coded by niche (*upper panel*) or cell type (*lower panel*), merged with a plot of the expression of the CCL2 and CXCL10 transcripts. ECs are a combination of ECs and LSECs 1, 2, and 3; Macrophages are a combination of KCs and MoMFs; and T-cells are a combination of T cells 1, 2, and 3. (*E*) Heatmap of inflammation- and fibrosis-related gene expression across each niche.

The spatial distance analysis further revealed that periportal endothelial subsets (ECs_and_LSECs_1/2) resided significantly closer to cholangiocytes than the pericentral ECs_and_LSECs_3 subset (Figure 10*E*). Periportal ECs exhibited a greater abundance of neighboring macrophages within a 20-μm radius (Figure 10*F*), suggesting greater opportunities for paracrine stimulation. The neighborhood (niche) analysis identified periportal niches enriched for inflammatory and fibrotic signatures that were characterized by the accumulation of MoMFs, inflammatory T cells, and elevated expression of the CCL2, CXCL10, ITGAM, CCR2, and COL1A1 transcripts (Figure 11*A*–*E*). These results indicate that periportal interaction zones represent spatially organized inflammatory–fibrotic microenvironments driven by IL1B–IL1R1 signaling.

**Figure 12:**
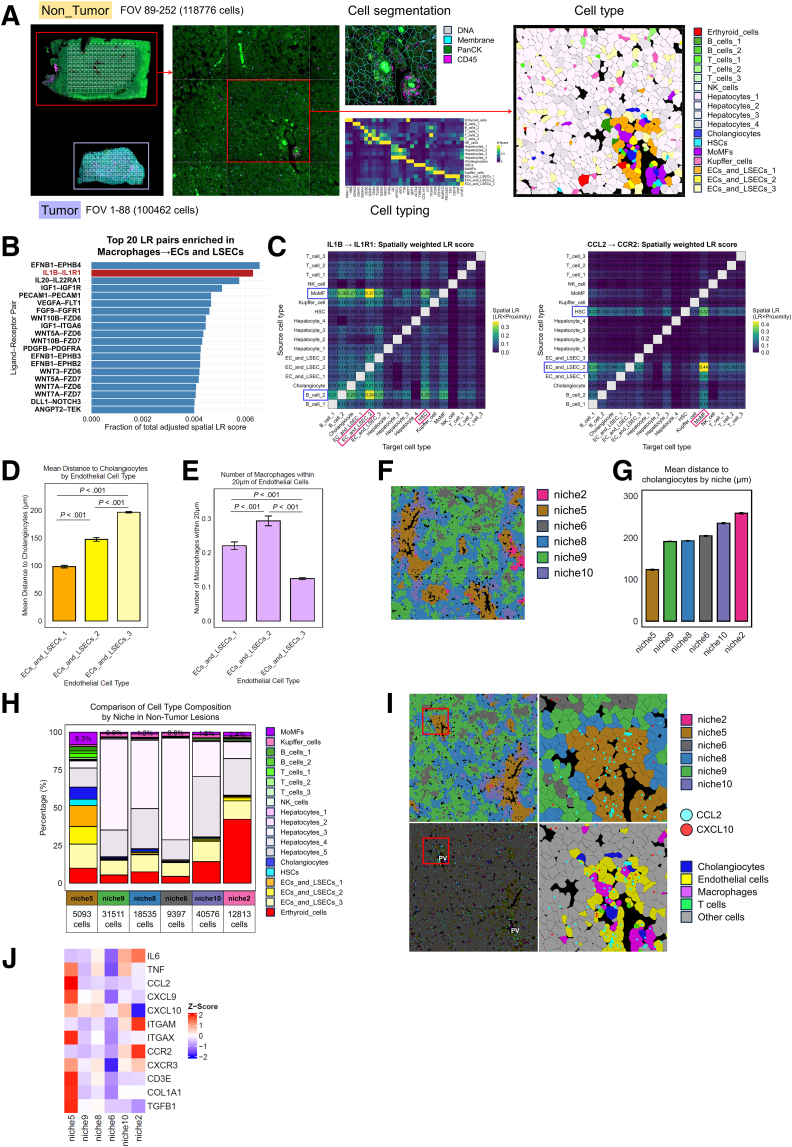
**Spatial transcriptomic validation of periportal inflammatory–fibrotic niches and macrophage–LSEC IL1B–IL1R1 interactions in a sample from an independent patient with MASH.** (*A*) Workflow for the analysis of liver tissues from a patient with MASH (CosMx_MASH_sample_2) using CosMx. (*B*) Top 20 LR pairs enriched in macrophage → EC/LSEC interactions based on spatial LR scores. (*C*) Heatmaps of spatially weighted LR scores. (*D*) Comparison of the shortest distance from each EC to the nearest cholangiocyte. (*E*) Comparison of the average number of macrophages within 20 μm of each EC. (*F*) Image of the FOV with the non-tumor regions color-coded by niche. (*G*) Bar plot of the mean distance to cholangiocytes by niche. (*H*) Bar plot showing the proportions of cell types in each niche of the non-tumor region. (*I*) Images of the FOV color-coded by niche (*upper panel*) or cell type (*lower panel*), merged with a plot of the expression of the CCL2 and CXCL10 transcripts. (*J*) A heatmap of inflammation- and fibrosis-related gene expression across each niche.

We next examined whether these findings were reproducible in an independent MASH sample (CosMx_MASH_sample_2) (Figure 12*A*). Consistent with the first sample, macrophage → endothelial/LSEC LR interactions were again strongly enriched, with IL1B–IL1R1 emerging as a top spatially weighted pair (Figure 12*B* and *C*). Chemokine-mediated signaling from EC/LSEC subsets toward MoMFs (eg, CCL2–CCR2) was also detected (Figure 12*C*). Periportal endothelial subsets were localized close to cholangiocytes and exhibited increased macrophage proximity (Figure 12*D* and *E*). The neighborhood analysis similarly identified periportal niches enriched for MoMFs and inflammatory–fibrotic gene signatures (Figure 12*F*–*J*). These results validate that macrophage-derived IL-1β stimulates periportal LSECs through IL1R1, reinforcing chemokine-driven inflammatory–fibrotic niches in MASH.

### Macrophage-derived IL-1β Induces CCL2 Expression in HSCs via JNK Activation

**Figure 13:**
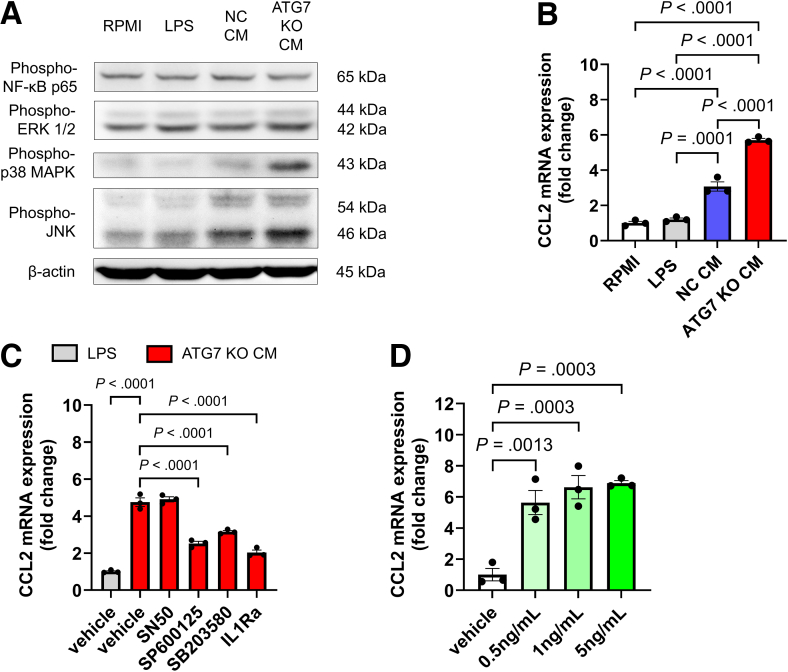
**Macrophage-derived IL-1β increases CCL2 expression in HSCs via JNK activation.** (*A*) Evaluation of the levels of NF-κB or MAPK-related proteins in LX-2 cells. (*B, C*) CCL2 gene expression in LX-2 cells treated with the control medium or THP-1 macrophage conditioned medium (n = 3/group). SN50, NF-κB inhibitor; SP600125, JNK inhibitor; SB203580, p38 MAPK inhibitor. (*D*) Evaluation of CCL2 expression in TMNK-1 cells after 48 hours of stimulation with recombinant human IL-1β (5 ng/mL) in the presence of SP600125 and SB203580 (n = 4/group).

Finally, based on the spatial transcriptomic findings that HSCs also expressed IL1R1 and exhibited elevated expression of CCL2 transcripts (Figure 10*D*, Figure 12*C*), we evaluated whether macrophage-derived IL-1β could directly induce chemokine production in HSCs. When LX-2 cells were cultured with conditioned medium from ATG7-deficient THP-1 macrophages, *CCL2* expression was markedly increased, accompanied by the activation of JNK and p38 MAPK signaling (Figure 13*A* and *B*). This induction was attenuated by IL1Ra and was significantly suppressed by JNK and p38 MAPK inhibition (Figure 13*C*), indicating that IL1β–IL1R1 signaling drives *CCL2* upregulation in HSCs via MAPK pathways. Furthermore, treatment with recombinant human IL-1β alone increased *CCL2* expression in a dose-dependent manner (Figure 13*D*). These data suggest that macrophage-derived IL-1β not only activates LSECs but also stimulates HSCs to produce CCL2, potentially amplifying immune cell recruitment and fibrotic remodeling in individuals with MASH.

## Discussion

In this study, we demonstrated that lipotoxic stress impairs autophagy in macrophages, accompanied by decreased TFEB activity, leading to increased secretion of IL-1β. This macrophage-derived IL-1β induced the expression of *CXCL10* and *CCL2* in LSECs through the activation of JNK and p38 MAPK pathways. In a MASH mouse model, genetic deletion of endothelial Il1r1 and systemic Cxcl10 deficiency ameliorated hepatic inflammation and fibrosis, and pharmacological inhibition of IL1R1 with anakinra produced similar therapeutic benefits. The spatial transcriptomic analysis further revealed that IL1B–IL1R1 signaling was enriched in periportal EC subsets that highly expressed chemokines and were located in close proximity to MoMFs. These findings indicate that macrophage–LSEC communication establishes periportal inflammatory–fibrotic niches that promote immune cell recruitment. In addition to LSECs, HSCs also responded to IL-1β by upregulating *CCL2*, suggesting that macrophage-derived IL-1β amplifies chemokine signaling through multiple NPC types.

With respect to the role of LSECs in liver disease, previous studies have focused primarily on adhesion molecules such as ICAM-1 and E-selectin, with few reports on chemokine release by LSECs themselves.^21^^,^^22^ In this study, we detected increased CXCL10 and CCL2 expression in LSECs during MASH. RNA sequencing (RNA-seq) of existing liver biopsy samples confirmed that the expression of these genes increased with MASH progression. Furthermore, we detected high *IL1R1* gene expression in ECs and HSCs in both mice and humans. Although CXCL10 expression is primarily induced by interferon (IFN)-γ stimulation and signals through the CXCR3 receptor, its expression showed little correlation with IFN-γ but was highly correlated with IL1B in liver biopsy samples from patients with MASLD (Figure 9*D*). *CXCL10* expression in the liver has been reported to play a role in monocyte and macrophage recruitment and MASH pathogenesis, but research on the role of LSEC-derived CXCL10 in MASH is limited. In our study, EC-specific IL1R1-KO mice with MASH presented reduced inflammatory cell infiltration due to suppressed chemokine expression in LSECs, which also led to decreased liver fibrosis. Although previous studies have demonstrated that hepatocyte-specific IL1R1 knockout improved MASH pathology,^23^ our study is the first to demonstrate the role of IL1R1 in ECs during MASH.

Studies using liver tissues from patients with MASLD/MASH have shown that CXCL10 and CCL2 expression correlate strongly with disease progression. Given that the IL-1β–IL-1R1 axis plays a crucial role in the expression of these genes, IL-1R1 inhibitors may be useful in suppressing MASH progression. The potential of targeting IL-1 for the treatment of liver diseases has previously been discussed.^24^^,^^25^ Anakinra, an IL-1R1 inhibitor, is a United States Food and Drug Administration (FDA)-approved treatment for conditions such as rheumatoid arthritis and Still’s disease. A multicenter, randomized, double-blind, placebo-controlled trial (the DASH trial) is currently evaluating the efficacy of anakinra for alcoholic steatohepatitis treatment.^26^ Anakinra has also been reported to improve insulin sensitivity and glucose tolerance in diabetic patients,^27^^,^^28^ raising prospects for its future application in MASLD, which is strongly associated with glucose intolerance. In our MASH mouse model, short-term administration of anakinra led to reduced hepatic inflammation and a trend toward attenuated fibrosis, accompanied by decreased expression of Ccl2, Itgam, and Tgfb1 in the liver tissue and reduced CD11b^+^ immune cell infiltration. These findings provide in vivo support that pharmacological IL-1R1 blockade can mitigate chemokine-mediated inflammatory responses and may help attenuate inflammatory–fibrotic progression in patients with MASH.

Evaluating autophagy in vivo is challenging, and there has been debate over whether autophagy is activated or suppressed in liver tissues from patients with MASH. We previously reported that fatty acid stimulation disrupts the late stages of autophagy in hepatocytes.^16^ In this study, we focused on TFEB, a key regulator of lysosomal biogenesis, during the late stages of autophagy. We observed a reduction in TFEB expression and autophagy suppression in THP-1 macrophages subjected to fatty acid overload. TFEB translocates to the nucleus in response to lysosomal stress, contributing to the maintenance of lysosomal function. However, the accumulation of chronic stress may lead to a reduction in TFEB activity over time, resulting in lysosomal dysfunction. This suggests a dual mechanism in which the nuclear translocation of TFEB serves as an initial adaptive response, whereas its subsequent downregulation contributes to chronic disease progression. Autophagy impairment associated with TFEB downregulation has also been reported in ethanol-induced liver injury, highlighting the importance of improving lysosomal function to slow the progression of steatohepatitis.^29^ Ezetimibe and curcumin have been reported to improve autophagy as TFEB activators,^30^, ^31^, ^32^ and the development of TFEB activators or autophagy-targeted therapies for the treatment of fatty liver diseases, including MASH, is anticipated in the future.

In conclusion, impaired autophagy in hepatic macrophages leads to increased IL-1β secretion, which activates periportal LSECs via IL1R1 signaling to induce the production of chemokines that recruit immune cells and amplify inflammatory–fibrotic remodeling in individuals with MASH. These results identify macrophage autophagy and IL-1β–IL1R1 signaling as actionable therapeutic targets that warrant further investigation in the treatment of MASH.

## Materials and Methods

### Mice

B6.129P2-Lyzs<tm1(cre)Ifo> mice (RBRC02302) (LysM-Cre mice) were provided by the RIKEN BRC through the National Bio-Resource Project of the MEXT, Japan. The Atg7^flox/flox^ mice were kindly provided by Professor Masaaki Komatsu of Juntendo University.^33^ These mice were crossed to produce LysM-Cre Atg7^flox/flox^ (Atg7^ΔMye^) mice.

Cdh5-Cre^ERT2^ mice were generously provided by Professor Yoshiaki Kubota in the Department of Anatomy at Keio University School of Medicine.^34^ B6.129(Cg)-Il1r1^<tm1.1Rbl>^/J (#028398) (Il1r1^flox/flox^) mice were purchased from Jackson Laboratory and crossed with Cdh5-Cre^ERT2^ mice to produce Cdh5-Cre^ERT2^ Il1r1^flox/flox^ (Il1r1^ΔEC^) mice. Tamoxifen (100 mg/kg/day; intraperitoneally) was administered once daily for 5 consecutive days to mice at 4 to 5 weeks of age to induce EC-specific deletion. B6.129S4-Cxcl10^tm1Adl^/J (#006087) (Cxcl10^-/-^) mice were purchased from Jackson Laboratory. All these genetically modified mice were backcrossed with C57BL/6J mice for more than 10 generations. As a control for Cxcl10^-/-^ mice, 4-week-old C57BL6/J mice were purchased from Jackson Laboratory Japan and were co-housed with Cxcl10^-/-^ mice.

The mice were fed either an ND (CRF-1; Oriental Yeast Company) or a WD for 12 to 48 weeks. The WD (D20092804) was custom-formulated by Research Diets, Inc to be high in fat (36.4% kcal, primarily from milk fat), cholesterol (1.21%), and sucrose-enriched carbohydrates (48.4% kcal).

We performed an experiment using the pharmacological inhibitor anakinra (Raleukin; Synonyms: Kineret; Anakinra; HY-108841; MedChemExpress) to evaluate the therapeutic effect of IL1R1 signaling on MASH progression. Mice were fed a WD for 24 weeks and were administered either phosphate buffered saline (PBS) or anakinra (10 mg/kg, intraperitoneally, 5 days per week) during the final 4 weeks. Body weight and serum ALT levels were measured prior to treatment initiation to ensure comparable baseline conditions between groups.

### Glucose Tolerance Test

The ND group and the WD group were fasted overnight before glucose tolerance testing (GTT). At 9:00 the next morning, a glucose solution (D-(+)-Glucose, D-G8270; Sigma-Aldrich) at a dosage of 1 g/kg body weight was injected intraperitoneally.^13^ Blood samples were collected from the tail vein at designated time points, and blood glucose levels were measured.

### Collection of Mouse Hepatocytes, Liver Macrophages, and LSECs

As we previously reported,^35^ the pronase–collagenase perfusion procedure was used to dissociate hepatocytes and NPCs into single-cell suspensions. The collected cells were suspended in Roswell Park Memorial Institute (RPMI) medium containing 2% FBS, filtered through a 70-μm mesh filter, and centrifuged at 500 × g for 10 minutes. After the supernatant was discarded, the pellet was resuspended, and the process was repeated. The pellet was then resuspended in RPMI medium and centrifuged at 50 × g for 10 minutes to separate the supernatant, which contained the NPCs, from the pellet containing hepatocytes. This separation process was repeated 2 to 4 times to increase the purity. Finally, the NPCs were collected from the pellet after centrifugation at 500 × g for 10 minutes. From the collected NPCs, LSECs were isolated using anti-CD146 MACS beads (130-092-007; Miltenyi Biotec), followed by isolation of liver macrophages using anti-F4/80 MACS beads (130-110-443; Miltenyi Biotec).^36^

### Flow Cytometry

After pronase and collagenase perfusion, NPCs isolated from mouse liver tissues were stained using the following antibodies: anti-CD45 (553080; BD Pharmingen), anti-Ly-6C (560593; BD Pharmingen), anti-Ly-6G (746448; BD Pharmingen), anti-CD11b (561689; BD Pharmingen), anti-F4/80 (565411; BD Pharmingen), and anti-7-AAD (559925; BD Pharmingen). The cells were then processed using a BD Cytofix/Cytoperm Fixation/Permeabilization Kit (554714; BD Biosciences) and stored at −80 °C. The frozen cells were thawed and stained for intracellular p62 expression with an anti-SQSTM1/p62 antibody [EPR4844] (ab109012; Abcam) and pre-adsorbed goat anti-rabbit IgG H&L (Alexa Fluor 647) (ab150083; Abcam). The stained cells were analyzed using a BD FACSCanto II Clinical Flow Cytometry System (BD Biosciences) and processed with FlowJo v10 software (BD Biosciences). Among the CD45^+^Ly6G^-^ cells, those characterized as CD11b^high^ F4/80^int^ were classified as MoMFs, and those characterized as CD11b^int^ F4/80^high^ were classified as KCs. The mean fluorescence intensity of p62 was measured for both cell types. The mice used in the study were Atg7^flox/flox^ mice, which served as wild-type controls.

We quantified the absolute number of hepatic MoMFs by calculating cell counts based on the total yield of isolated NPCs and flow cytometric frequencies using the following formula:

Number of isolated MoMFs = Total live NPCs × (%CD45^+^ of live) × (%MoMF of CD45^+^).

### Single-cell RNA-seq Analysis

The mouse livers were perfused with pronase and collagenase, followed by centrifugation at 50 × g for 10 minutes to separate hepatocytes and NPCs. The cells were mixed at a ratio of approximately 1:4 (hepatocytes to NPCs) to improve the analysis quality to focus on the function of NPCs. Single-cell analysis was performed using a BD Rhapsody Express System (BD Biosciences) according to the manufacturer’s protocol. Rhapsody WTA libraries were sequenced on a DNBSEQ-G400RS (MGIF-400; MGI Tech Co, Ltd), and raw sequence reads were demultiplexed to generate fastq files for each sample using SplitBarcode v2.0.0 (MGI Tech Co, Ltd). Single-cell RNA data were analyzed using Seurat (version 5) in R (version 4.3.3).

### Histological Analysis

Paraffin-embedded sections of mouse liver fixed in 10% formalin were prepared and stained with hematoxylin and eosin (H&E) and Sirius Red. Sirius Red staining was performed using a Picrosirius Red Staining Kit (24901; Polysciences, Inc). To evaluate inflammatory cell infiltration, we used a VECTASTAIN ABC-HRP Kit and peroxidase (rat immunoglobulin G) (PK-4004; Vector Laboratories) and stained the samples with a rabbit anti-CD3 epsilon antibody (ab5690; Abcam) and a rabbit anti-CD11b antibody [EPR1344] (ab133357; Abcam). The stained sections were imaged using a VS200 Research Slide Scanner (EVIDENT), and positive areas were quantified using HALO software (Indica Labs).

### Cell Culture

THP-1 cells (JCRB0112), TMNK-1 cells (JCRB1564), and LX-2 cells (JCRB1569) were purchased from the JCRB Cell Bank. TMNK-1 cells and LX-2 cells were cultured in Dulbecco’s modified Eagle’s medium (DMEM) (D6429; Sigma-Aldrich) supplemented with 10% FBS (16000044; Thermo Fisher Scientific) and 1% antibiotics (15240062; Thermo Fisher Scientific). THP-1 cells were cultured in RPMI medium (R8758; Sigma-Aldrich) supplemented with the same supplements at 37°C and 5% CO_2_. THP-1 cells were stimulated with 100 ng/mL phorbol 12-myristate 13-acetate (PMA) (P1585; Sigma-Aldrich) for 48 hours to induce their differentiation into macrophages.^37^ After differentiation, the cells were washed 3 times with PBS, fresh RPMI medium was added, and the cells were incubated overnight before being used in subsequent experiments.

### Plasmids

Using lentiCRISPR v2-Blast (Addgene, 83480) as a template, we constructed a lentiCRISPR ATG7 KO plasmid and a lentiCRISPR negative control (NC) plasmid using the CRISPR–Cas9 system. The guide RNA sequences for ATG7 were as follows: forward, 5′-CACCGAGAAGAAGCTGAACGAGTAT-3′; reverse, 5′-AAACATACTCGTTCAGCTTCTTCTC-3′. The guide RNA sequences for the negative control were as follows: forward, 5′-CACCGTGGCGGCCCAAACTTAACAC-3′; reverse, 5′-AAACGTGTTAAGTTTGGGCCGCCAC-3′. The constructed plasmids were cotransfected with the PAX2 plasmid (Addgene, #12260) and the VSVG plasmid (Addgene, #8454) into HEK293 cells to generate lentiviruses. The culture supernatant containing the lentiviruses was then applied to THP-1 cells, followed by selection with blasticidin (Sigma–Aldrich, B12150). After selection, single-cell clones were generated by limiting dilution and cultured. The KO efficiency was confirmed by Western blotting.

### Enzyme-linked Immunosorbent Assay

THP-1 macrophages were pretreated for 1 hour with 50 μM Z-YVAD-FMK (S8507; Selleck, Houston, TX) before stimulation. The cells were subsequently stimulated for 24 hours with 25 ng/mL LPS (L2630; Sigma–Aldrich) in combination with bovine serum albumin (BSA), 400 μM PA, 50 nM BFM, or each agent alone. The culture supernatants were then collected. The contents of IL-1β and caspase-1 in the culture supernatants were measured using a Human IL-1 beta/IL-1F2 DuoSet enzyme-linked immunosorbent assay (ELISA) (DY201-05; R&D Systems) and a Human caspase-1/ICE Quantikine ELISA Kit (DCA100; R&D Systems), respectively.

### Western Blotting

The protocols for sample preparation, electrophoresis, transfer, luminescence detection, and imaging of the cell lines or liver tissue were performed as previously described.^38^ The list of antibodies used is provided in Table 1. Nuclear proteins were extracted using a Nuclear Extraction Kit (Abcam, ab113474) according to the manufacturer’s protocol.

**Table 1:** List of Antibodies Used for Western Blotting

Purchased from	Cat. No.	Protein	Product name	Dilution	Source
Sigma	A5316	β-Actin	Monoclonal anti-β-actin antibody produced in mouse	1:3000	Mouse
Cell Signaling Technology	#13435	Lamin B1	Lamin B1 (D9V6H) rabbit mAb	1:1000	Rabbit
Cell Signaling Technology	#2631	Atg7	Atg7 antibody	1:1000	Rabbit
Cell Signaling Technology	#5144	SQSTM1/p62	SQSTM1/p62 antibody	1:1000	Rabbit
Cell Signaling Technology	#2775	LC3B	LC3B antibody	1:1000	Rabbit
Cell Signaling Technology	#8242	NF-κB p65	NF-κB p65 (D14E12) XP rabbit mAb	1:1000	Rabbit
Cell Signaling Technology	#3033	Phospho-NF-κB p65	Phospho-NF-κB p65 (Ser536) (93H1) rabbit mAb	1:1000	Rabbit
Cell Signaling Technology	#9252	JNK	SAPK/JNK antibody	1:1000	Rabbit
Cell Signaling Technology	#9251	Phospho-JNK	Phospho-SAPK/JNK (Thr183/Tyr185) antibody	1:1000	Rabbit
Cell Signaling Technology	#9212	p38 MAPK	p38 MAPK antibody	1:1000	Rabbit
Cell Signaling Technology	#4511	Phospho-p38 MAPK	Phospho-p38 MAPK (Thr180/Tyr182) (D3F9) XP rabbit mAb	1:1000	Rabbit
Cell Signaling Technology	#4695	Erk1/2	p44/42 MAPK (Erk1/2) (137F5) rabbit mAb	1:1000	Rabbit
Cell Signaling Technology	#4370	Phospho-Erk1/2	Phospho-p44/42 MAPK (Erk1/2) (Thr202/Tyr204) (D13.14.4E) XP rabbit mAb	1:1000	Rabbit
Cell Signaling Technology	#12242	IL-1β	IL-1β (3A6) mouse mAb	1:1000	Mouse
Cell Signaling Technology	#4240	TFEB	TFEB antibody	1:1000	Rabbit
Cell Signaling Technology	#37681	Phospho-TFEB	Phospho-TFEB (Ser211) (E9S8N) rabbit mAb	1:1000	Rabbit
Cell Signaling Technology	#32361	TFEB	TFEB (D4L2P) rabbit mAb	1:1000	Rabbit

### Quantitative Real-time Reverse-transcription Polymerase Chain Reaction

Total RNA was extracted from cells or tissues and reverse transcribed to complementary DNA (cDNA) as previously described.^16^ Messenger RNA (mRNA) expression was measured using quantitative real-time reverse-transcription polymerase chain reaction (qRT-PCR) with Thunderbird qPCR Master Mix (Toyobo) and TaqMan probes (Thermo Fisher Scientific). The expression of the target genes was normalized to that of β-actin and expressed as fold changes relative to those of the control group. The probes used are listed in Table 2.

**Table 2:** List of TaqMan Probes Used for qRT-PCR

Species	Gene	Assay ID	Species	Gene	Assay ID
Mouse	Actb	Mm02619580_g1	Human	ACTB	Hs01060665_g1
	Il1b	Mm00434228_m1		CCL2	Hs00234140_m1
	Ccl2	Mm00441242_m1		CXCL9	Hs00171065_m1
	Cxcl9	Mm00434946_m1		CXCL10	Hs00171042_m1
	Cxcl10	Mm00445235_m1		IL1B	Hs01555410_m1
	Cd68	Mm03047343_m1			
	Ccr2	Mm99999051_gH			
	Itgam	Mm00434455_m1			
	Itgax	Mm00498698_m1			
	Cxcr3	Mm99999054_s1			
	Il1r1	Mm00434235_m1			
	Col1a1	Mm00801666_g1			
	Tgfb1	Mm01178820_m1			
	Alb	Mm00802090_m1			
	Lyve1	Mm00475056_m1			
	Adgre1	Mm00802529_m1			
	Clec4f	Mm00443934_m1			
	Atg7	Mm00512209_m1			
	Sqstm1	Mm00448091_m1			
	Fos	Mm00487425_m1			
	Jun	Mm07296811_s1			

qRT-PCR, quantitative real-time reverse-transcription polymerase chain reaction.

### Evaluation of Autophagy

PA was prepared as previously described.^39^ THP-1 macrophages were stimulated with various concentrations of PA for 24 hours, followed by a 2-hour treatment with or without 125 nM BFM A1 (BioViotica). Proteins were then collected and analyzed by Western blotting. The autophagic flux index was calculated as the ratio of the LC3-II protein level in the presence of BFM A1 to the level in its absence.

### Conditioned Medium Experiments

Culture supernatants from THP-1 macrophages (negative control or ATG7-KO) stimulated with 25 ng/mL LPS were used as conditioned media. This conditioned medium was then mixed with the supernatant of TMNK-1 cells at a 50% volume ratio. After 24 hours, proteins and RNA were collected for analysis. To investigate the effects on the NF-κB and MAPK pathways, TMNK-1 cells were pretreated with various inhibitors for 1 hour, followed by the addition of culture supernatants from LPS-stimulated ATG7 KO THP-1 macrophages. After 24 hours, RNA was harvested from the cells. The inhibitors used were as follows: the NF-κB inhibitor SN50 (HY-P0151; MedChemExpress), the JNK inhibitor SP600125 (HY-12041; MedChemExpress), the p38 MAPK inhibitor SB 203580 (HY-10256; MedChemExpress), and the IL-1R antagonist (IL-1Ra) (093-05991; FUJIFILM Wako Pure Chemical Corporation). To assess the effects of IL-1β, TMNK-1 cells were treated with 5 ng/mL recombinant human IL-1 beta protein (ab9617; Abcam), and RNA was harvested from the cells.

In parallel, conditioned media from THP-1 macrophages stimulated with 25 ng/mL LPS were also added to LX-2 cells at a 50% volume ratio. After 48 hours of incubation, RNA was collected from the cells for analysis.

### Clinical Samples From Patients With MASLD/MASH

Surgically resected specimens from patients with MASLD/MASH were obtained with approval from the Institutional Review Board at Osaka University Hospital (IRB No. 15267).

### RNA-seq of Patient Liver Biopsy Samples

We previously reported and published the results of liver tissue RNA-seq from 98 patients with NAFLD/nonalcoholic steatohepatitis (NASH) (GSE167523).^20^ Based on the clinical data, we confirmed that NAFLD corresponds to non-MASH and that NASH corresponds to MASH, and we reanalyzed the raw count data using iDEP_2.01.

### Spatial Transcriptome Analysis

Formalin-fixed paraffin-embedded (FFPE) liver tissue sections from patients with MASH-related HCC were cut at a 5-μm thickness and mounted onto CosMx-compatible slides. Samples were prepared according to the manufacturer’s protocol (NanoString Technologies). Anti-B2M/CD298, anti-PanCK, anti-CD45, and anti-CD3 antibodies, together with 4′,6-diamidino-2-phenylindole (DAPI), were used as morphological markers. Gene expression was profiled using the CosMx Human Universal Cell Characterization RNA Panel (1000-plex).

Cell segmentation was performed for all cells within the available fields of view (FOVs) on a single slide containing both tumor and non-tumor regions. Preliminary analysis, cell typing, and microenvironment (niche) annotations were conducted using the AtoMx Spatial Informatics Platform (NanoString). Cell type assignments were guided by the Human Liver RDS reference from the CellProfileLibrary and were manually curated. Transcript coordinate visualization and cell type/niche rendering were performed within AtoMx, and resulting images were exported.

Cell metadata, expression matrices, transcript coordinates, FOV coordinates, cell type annotations, and niche classifications were exported as a Seurat object for downstream analysis in R. For this study, analyses were restricted to non-tumor regions. Pairwise intercellular distances were computed from XY coordinate data using the Euclidean distance, and the minimum distance between specified cell type pairs was extracted.

We comprehensively assessed LR interactions by extracting 364 one-to-one LR pairs from the CellChat database present within the 1000-plex panel. Spatial LR scores were computed by integrating LR expression levels with directional spatial proximity between cell types. Interactions between identical cell types (self-self pairs) were excluded to focus on intercellular rather than autocrine signaling.

All computational workflows, including Seurat processing, cell type annotations, directional spatial proximity scoring, and spatial LR weighting, are openly available on Zenodo together with the Seurat objects, metadata, and expression matrices (https://doi.org/10.5281/zenodo.14921039 for data; https://doi.org/10.5281/zenodo.17532914 for the analysis code and documentation), ensuring full reproducibility.

### Statistical Analyses

Statistical analyses were performed using GraphPad Prism 10.1.1 (GraphPad Software). For analyses not specifically mentioned, the following statistical tests were used:

For comparisons between 2 groups in in vitro experiments, assuming normality, an unpaired Student’s *t*-test was used. For comparisons involving 3 or more groups, 1-way analysis of variance (ANOVA) was followed by Tukey’s post hoc test for multiple comparisons. When each group was compared with the control group, Dunnett’s multiple comparisons test was applied. For comparisons involving multiple groups with 2 factors, such as the presence or absence of LPS and PA, 2-way ANOVA was performed, followed by Šídák’s multiple comparisons test. For in vivo studies, normality was first assessed using the Shapiro-Wilk test for all data. If normality was confirmed, the same statistical tests used for the in vitro experiments, such as Student’s *t*-test and 1-way ANOVA, were applied. If normality was not confirmed, nonparametric tests were used, with the Mann-Whitney *U* test used for comparisons between 2 groups. All bar plots in the figures are presented as the means ± standard errors of the mean (SEM) unless otherwise noted.

## Supplementary Material

Supplementary Material

Extended PDF
