# Supplementary material for: Liver Sinusoidal Endothelial Cells Promote Metabolic Dysfunction-associated Steatohepatitis Progression via Interleukin-1R1-mediated Chemokine Production Induced by Macrophage-derived Interleukin-1β
Source: Cell Mol Gastroenterol Hepatol. 2025 Dec 2;20(4):101698. doi: 10.1016/j.jcmgh.2025.101698 (PMC12865640; doi:10.1016/j.jcmgh.2025.101698)
Supplement: Supplementary Material [file mmc1.pdf]

Figure 1C

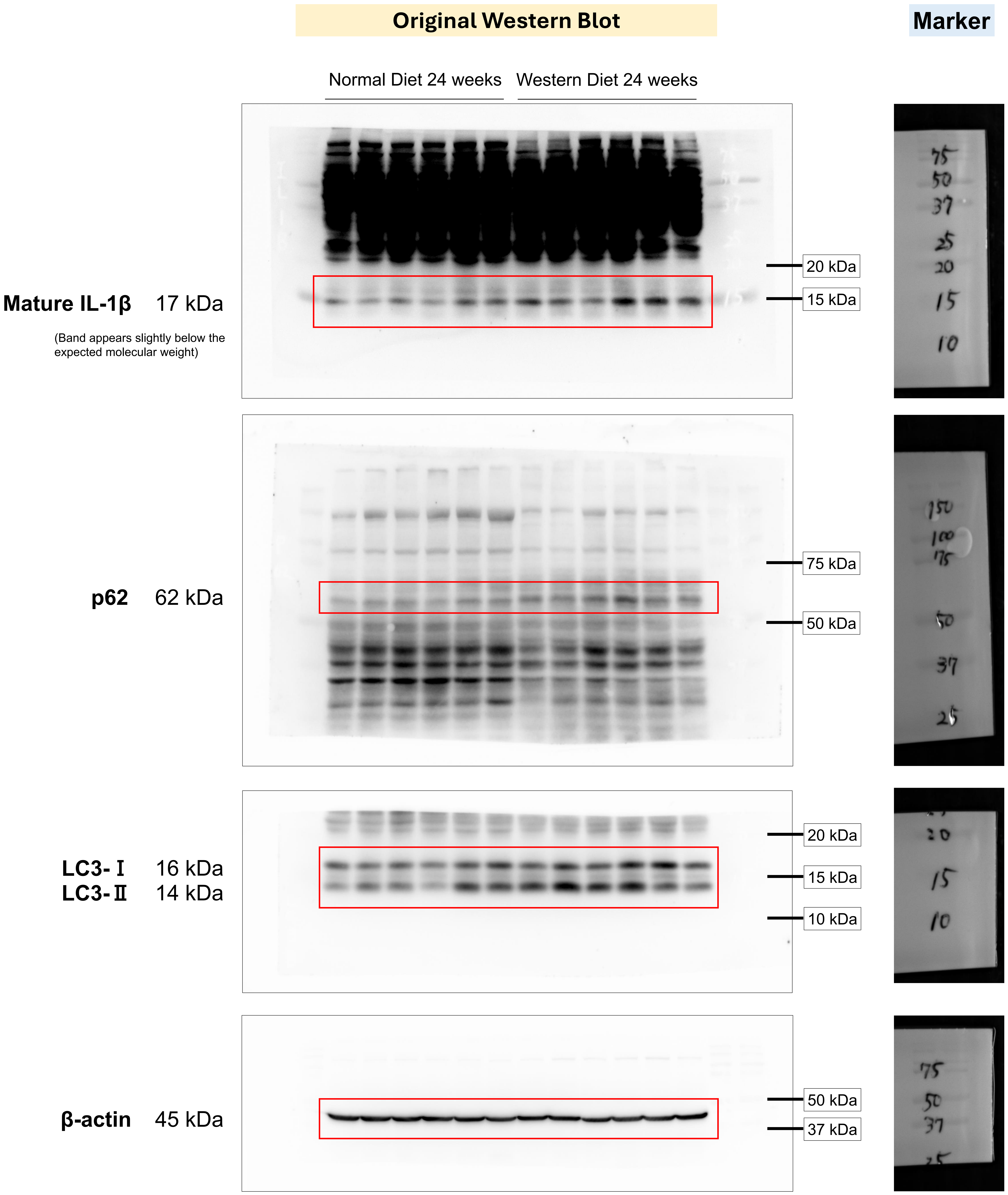

Original Western Blot
Marker
Normal Diet 24 weeks
Western Diet 24 weeks
Mature IL-1β
17 kDa
(Band appears slightly below the expected molecular weight)
20 kDa
15 kDa
p62
62 kDa
75 kDa
50 kDa
LC3-Ⅰ
16 kDa
LC3-Ⅱ
14 kDa
20 kDa
15 kDa
10 kDa
β-actin
45 kDa
50 kDa
37 kDa

Figure 2C

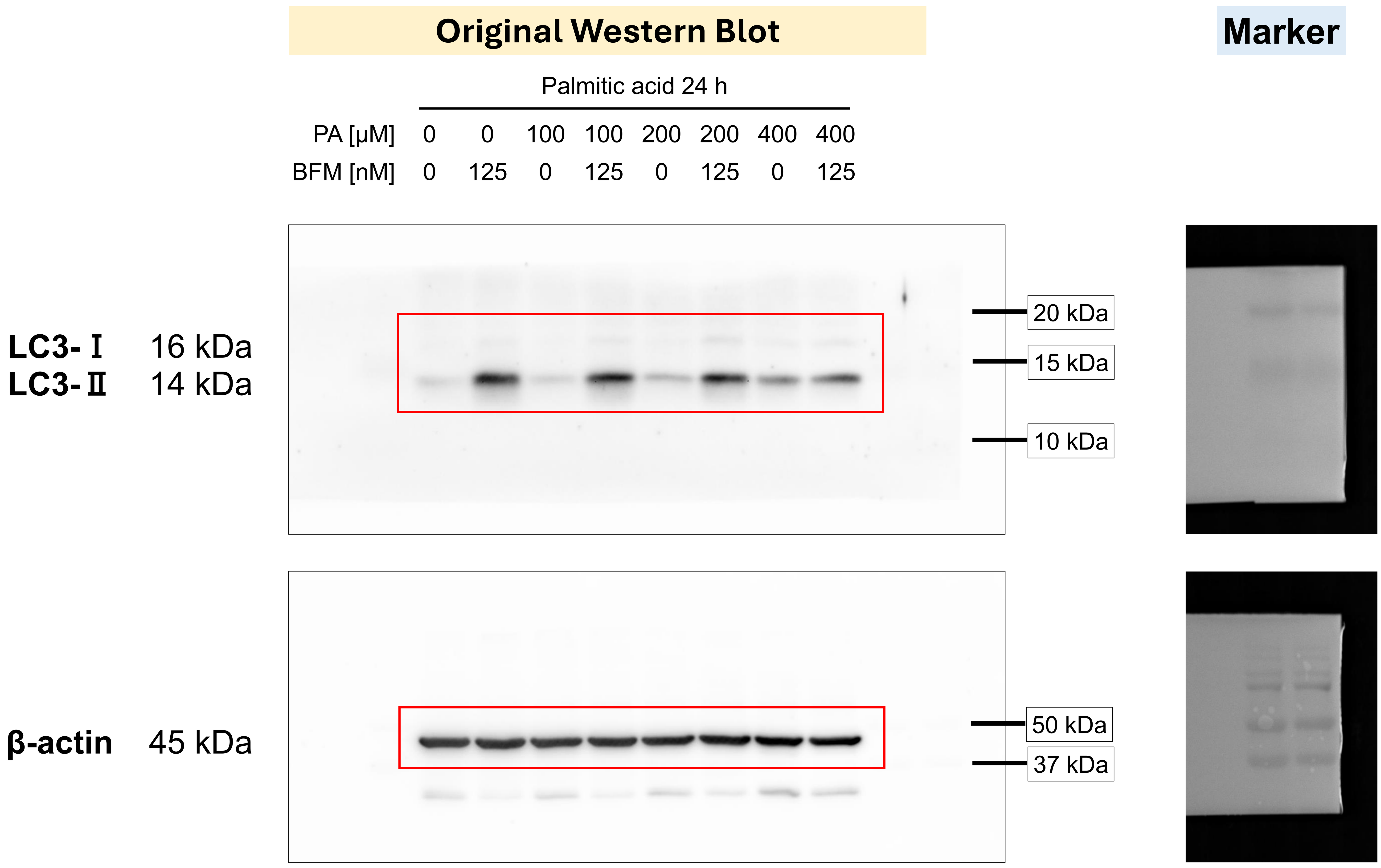

Original Western Blot
Marker
Palmitic acid 24 h
PA [μM] 0 0 100 100 200 200 400 400
BFM [nM] 0 125 0 125 0 125 0 125
LC3-Ⅰ 16 kDa
LC3-Ⅱ 14 kDa
20 kDa
15 kDa
10 kDa
β-actin 45 kDa
50 kDa
37 kDa

Figure 2D

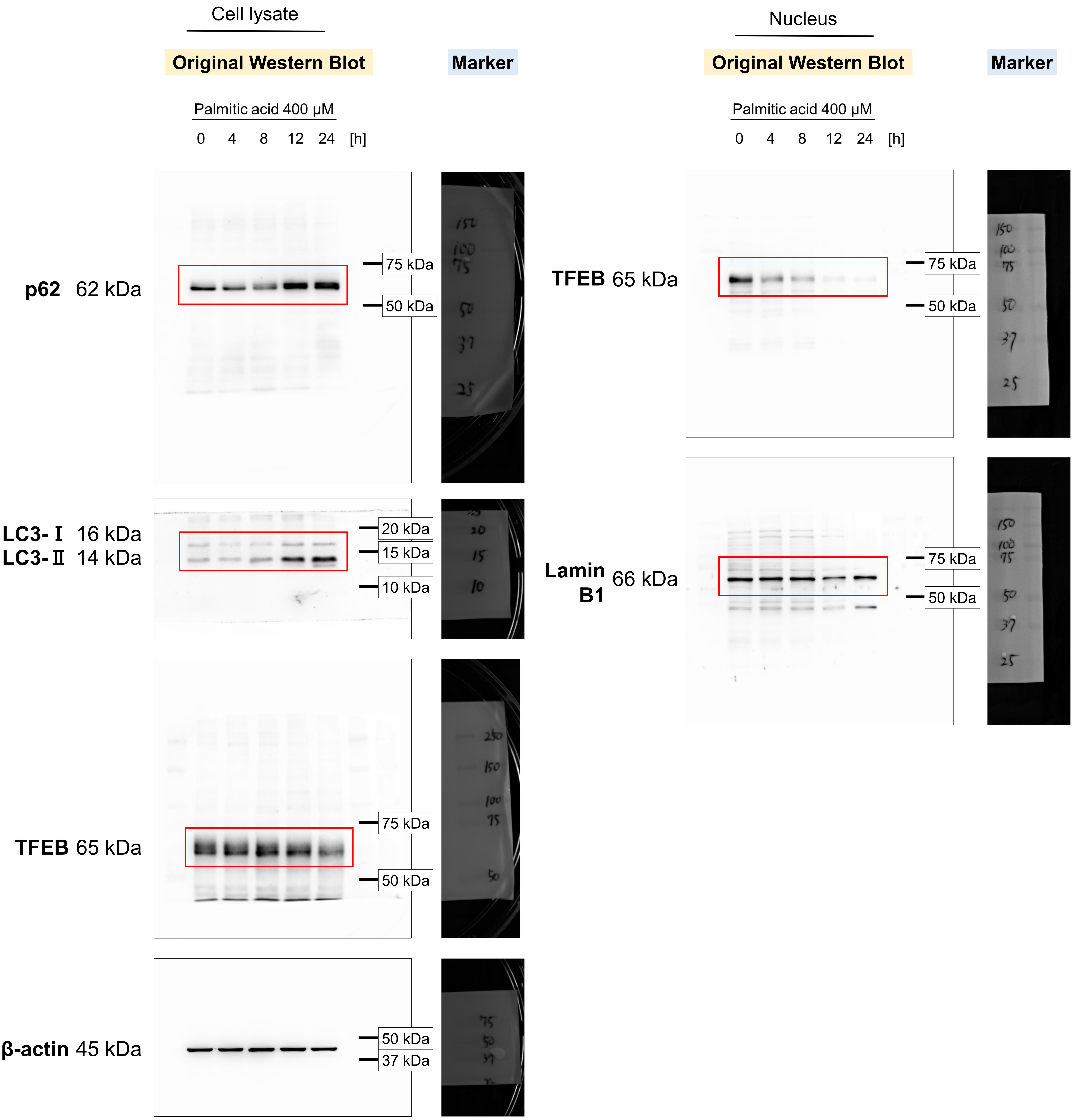
Cell lysate
Original Western Blot
Marker
Palmitic acid 400 μM
0 4 8 12 24 [h]
p62 62 kDa
75 kDa
50 kDa
LC3-Ⅰ 16 kDa
LC3-Ⅱ 14 kDa
20 kDa
15 kDa
10 kDa
TFEB 65 kDa
75 kDa
50 kDa
β-actin 45 kDa
50 kDa
37 kDa
Nucleus
Original Western Blot
Marker
Palmitic acid 400 μM
0 4 8 12 24 [h]
TFEB 65 kDa
75 kDa
50 kDa
Lamin B1 66 kDa
75 kDa
50 kDa

Figure 4A

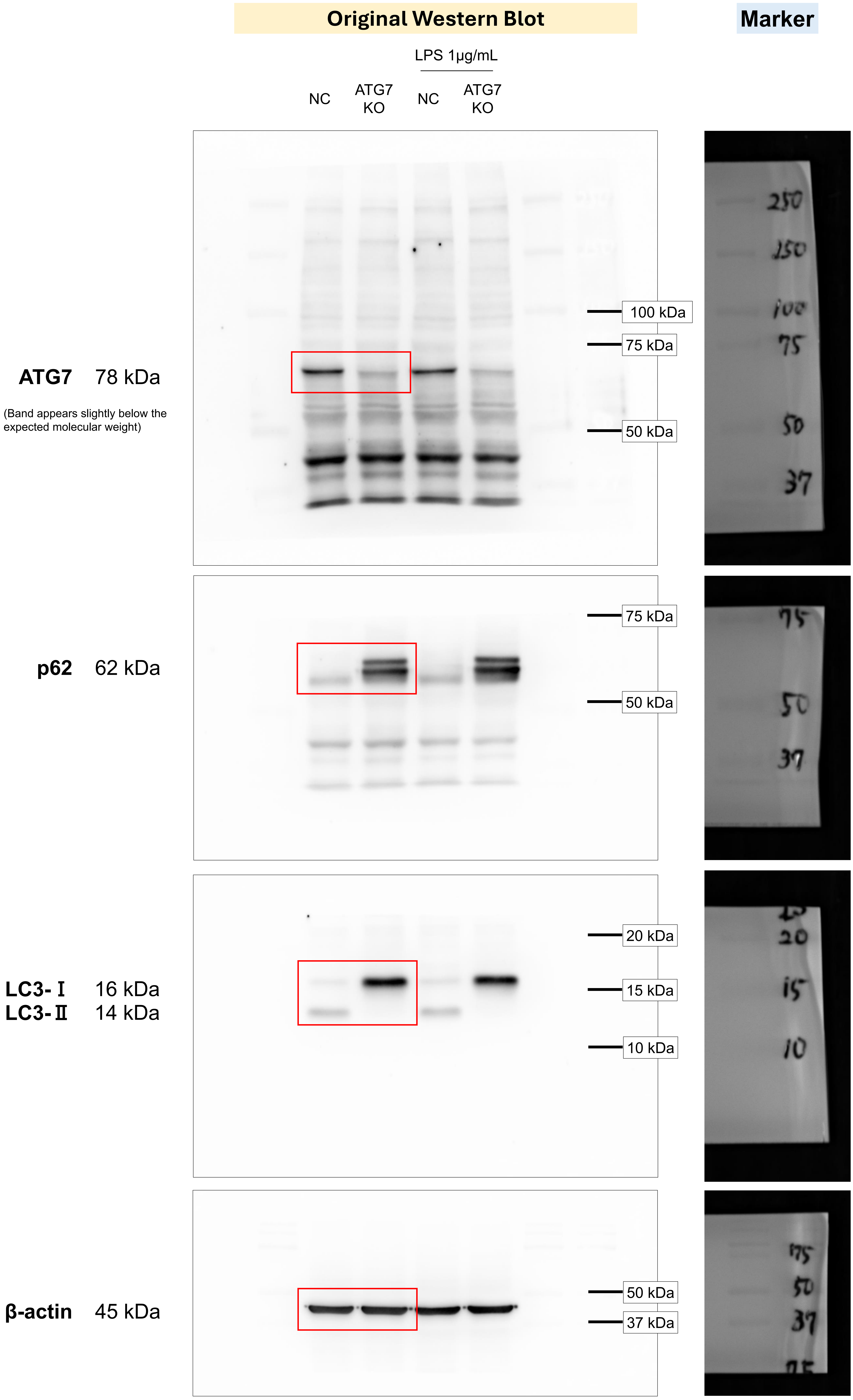
Original Western Blot
Marker
LPS 1μg/mL
NC
ATG7 KO
NC
ATG7 KO
ATG7 78 kDa
(Band appears slightly below the expected molecular weight)
100 kDa
75 kDa
50 kDa
p62 62 kDa
75 kDa
50 kDa
LC3- I 16 kDa
LC3- II 14 kDa
20 kDa
15 kDa
10 kDa
β-actin 45 kDa
50 kDa
37 kDa

Figure 4D

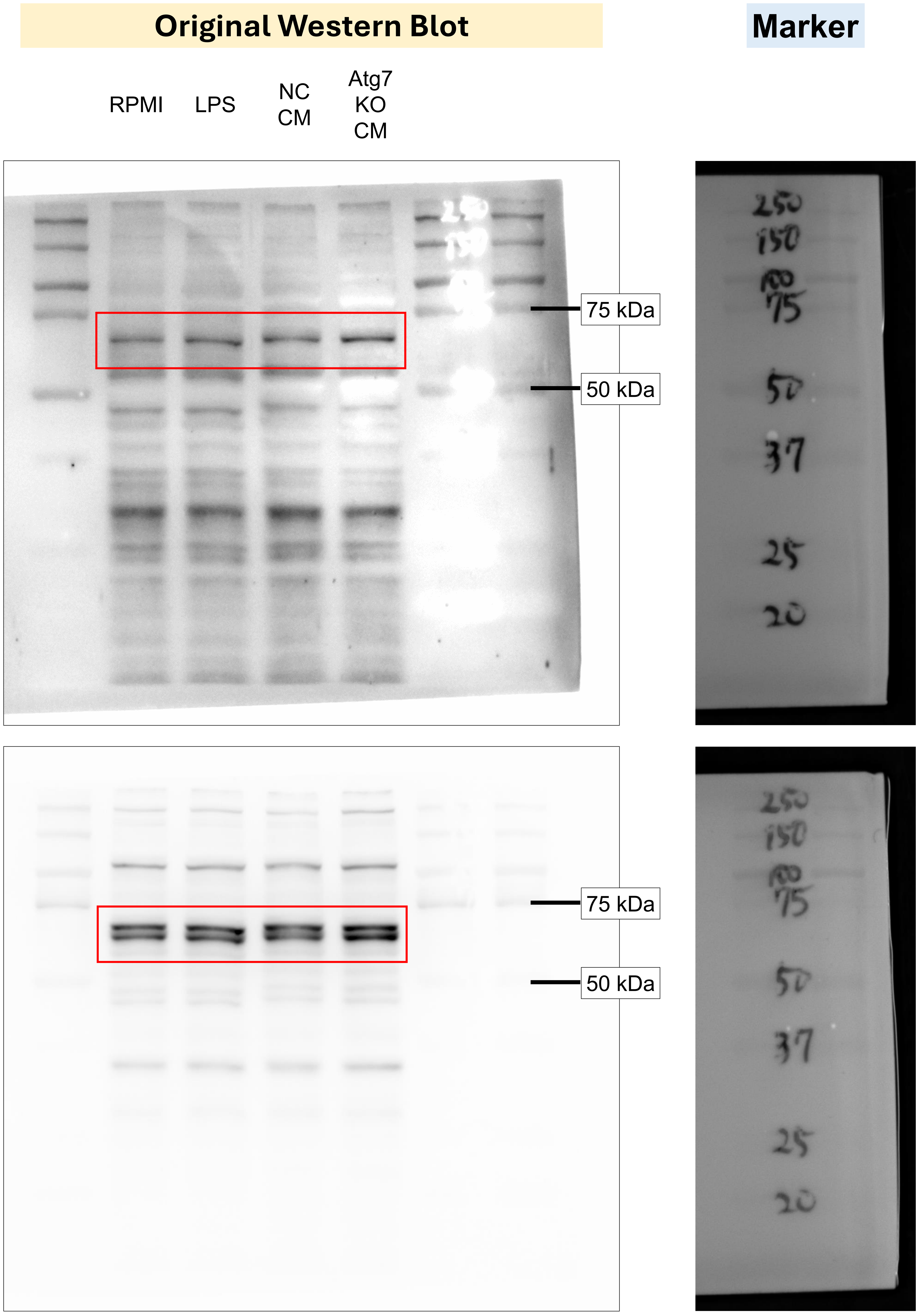

Phospho-NF-κB p65 65 kDa

NF-κB p65 65 kDa

The membrane was stripped and reprobed with anti–NF-κB p65 after detection of phospho–NF-κB p65.

Figure 4D

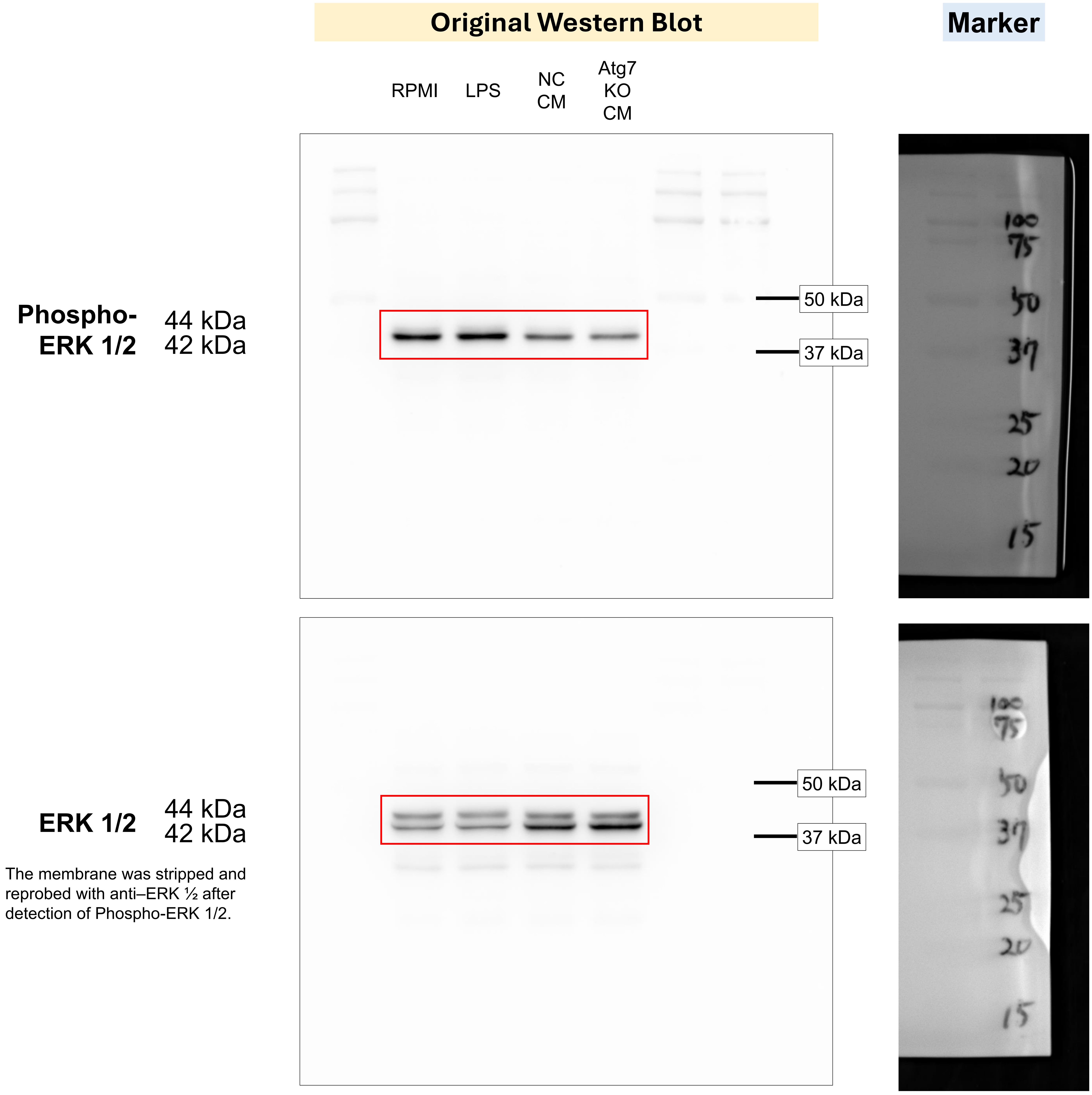
Original Western Blot
Marker
RPMI
LPS
NC CM
Atg7 KO CM
Phospho-ERK 1/2
44 kDa
42 kDa
50 kDa
37 kDa
100
75
50
37
25
20
15
ERK 1/2
44 kDa
42 kDa
50 kDa
37 kDa
100
75
50
37
25
20
15
The membrane was stripped and reprobed with anti–ERK ½ after detection of Phospho-ERK 1/2.

Figure 4D

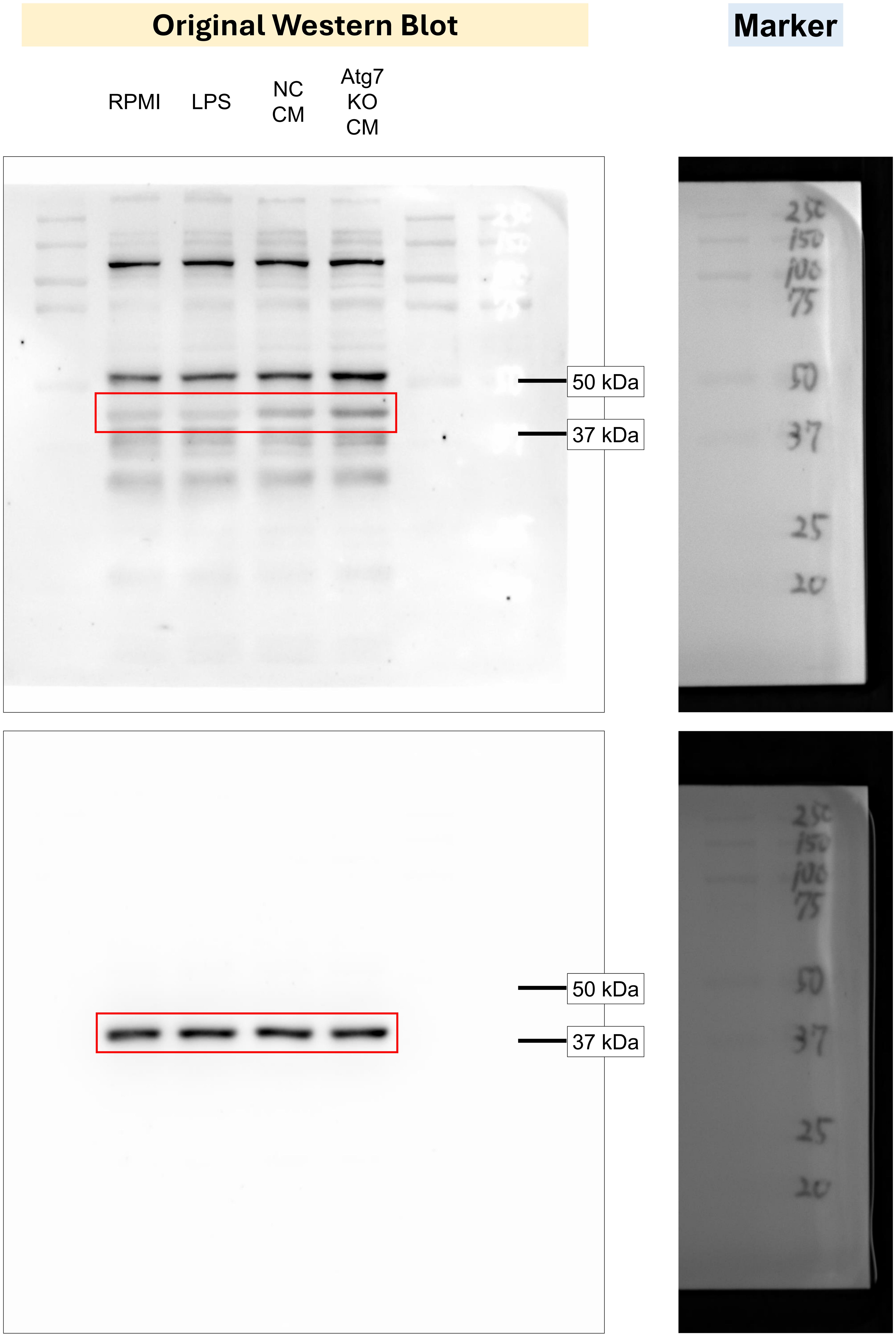

Phospho-p38 MAPK 43 kDa

p38 MAPK 40 kDa

The membrane was stripped and reprobed with p38 MAPK after detection of p38 Phospho-MAPK.

Figure 4D

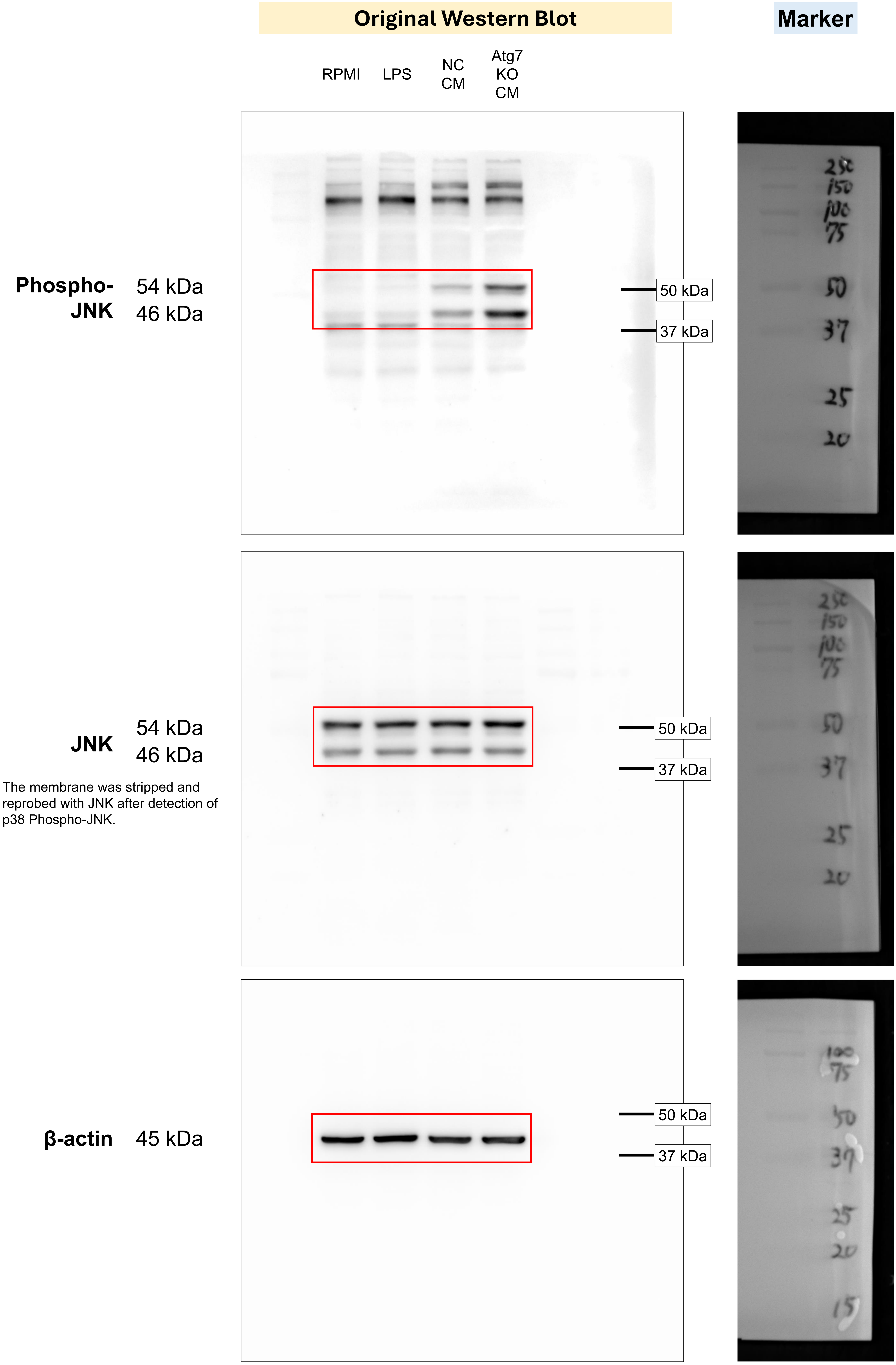
Original Western Blot
Marker
RPMI
LPS
NC CM
Atg7 KO CM
Phospho-JNK
54 kDa
46 kDa
50 kDa
37 kDa
JNK
54 kDa
46 kDa
The membrane was stripped and reprobed with JNK after detection of p38 Phospho-JNK.
50 kDa
37 kDa
β-actin
45 kDa
50 kDa
37 kDa

Figure 5A

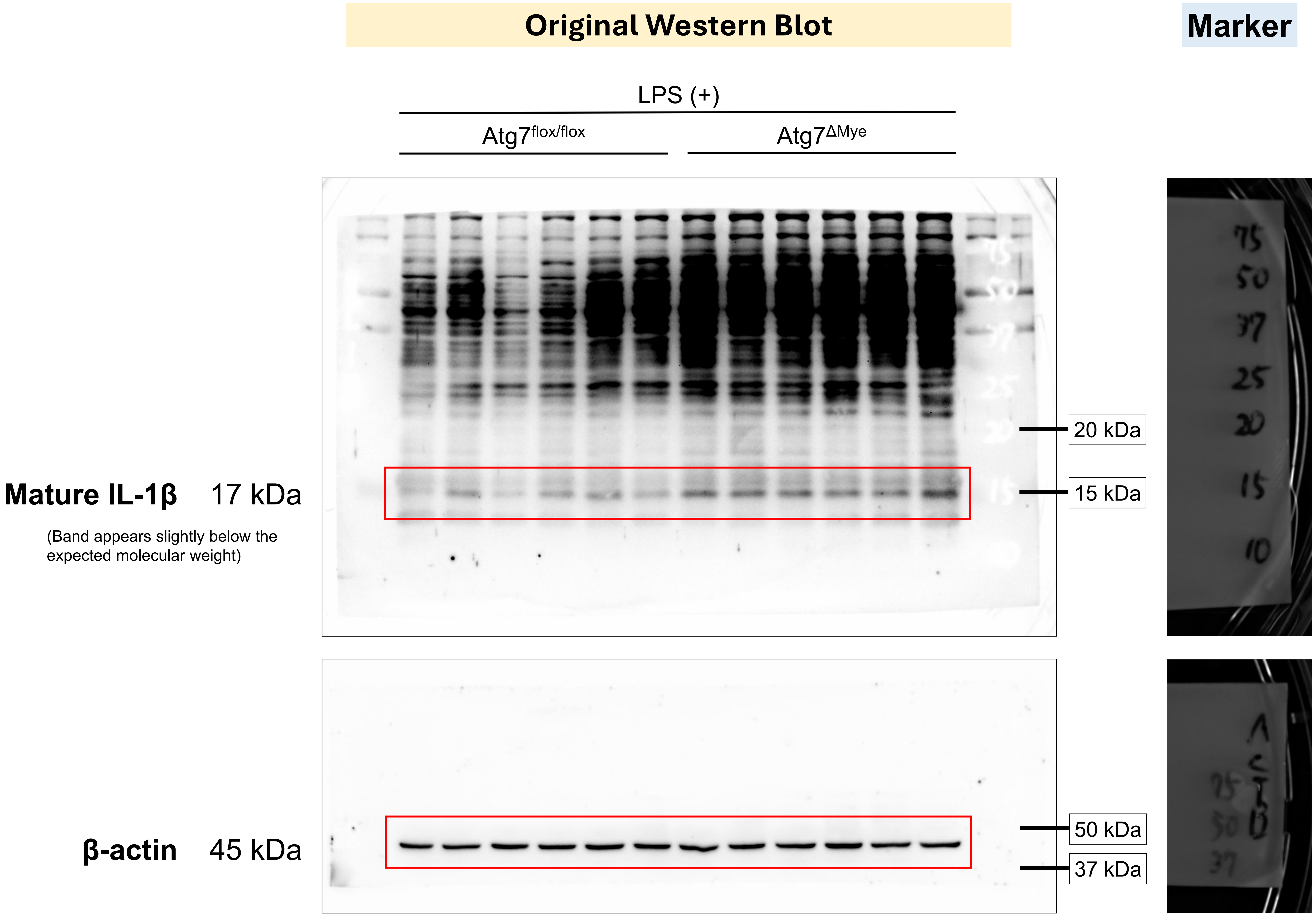
Original Western Blot
Marker
LPS (+)
Atg7flox/flox
Atg7ΔMye
Mature IL-1β 17 kDa
(Band appears slightly below the expected molecular weight)
20 kDa
15 kDa
β-actin 45 kDa
50 kDa
37 kDa
75
50
37
25
20
15
10

Figure 13A

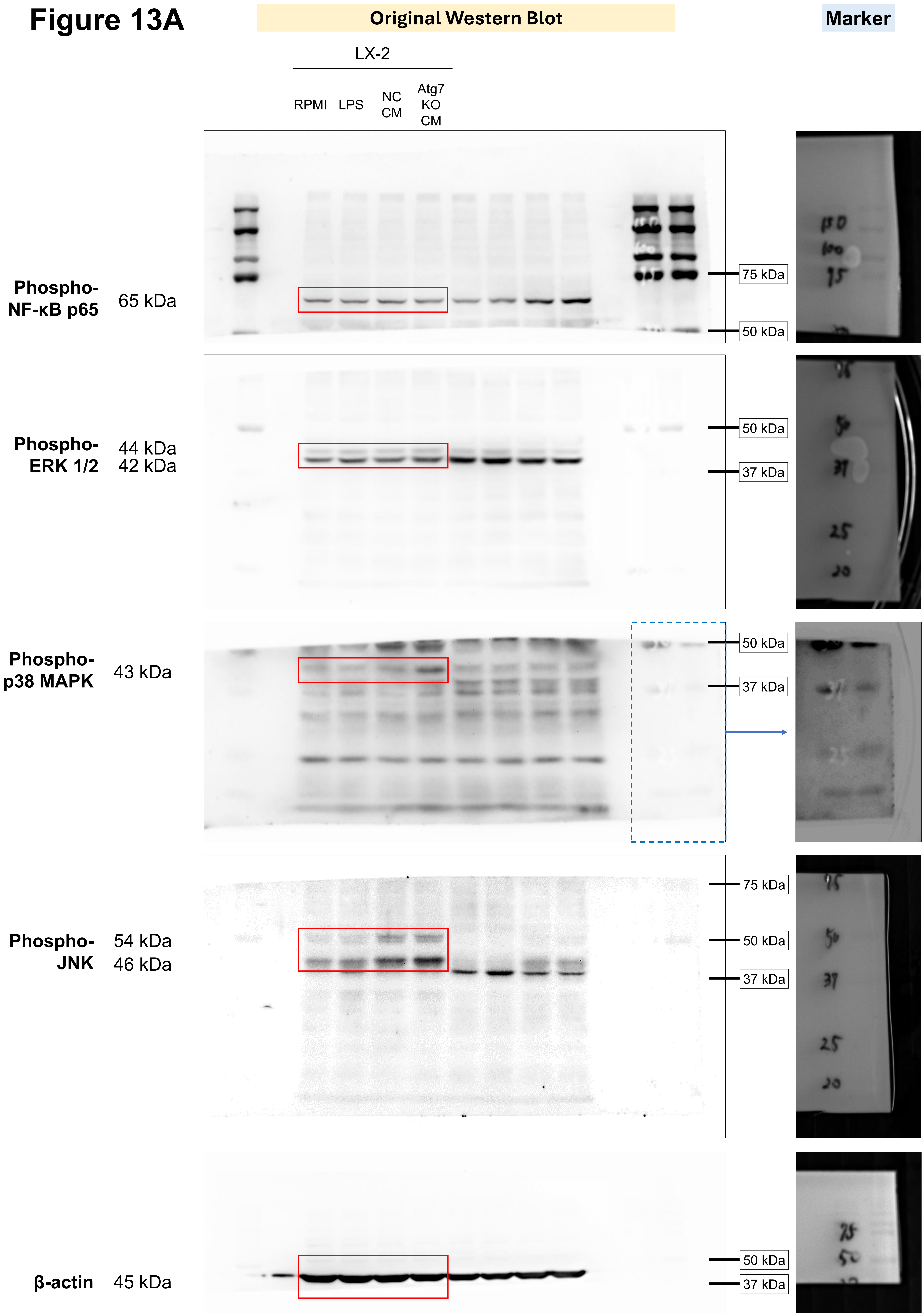
Original Western Blot
Marker
LX-2
RPMI
LPS
NC CM
Atg7 KO CM
Phospho-NF-κB p65
65 kDa
75 kDa
50 kDa
Phospho-ERK 1/2
44 kDa
42 kDa
50 kDa
37 kDa
Phospho-p38 MAPK
43 kDa
50 kDa
37 kDa
Phospho-JNK
54 kDa
46 kDa
75 kDa
50 kDa
37 kDa
β-actin
45 kDa
50 kDa
37 kDa
